# SEmHuS: a semantically embedded humanitarian space

**DOI:** 10.1186/s41018-023-00135-4

**Published:** 2023-03-07

**Authors:** Aladdin Shamoug, Stephen Cranefield, Grant Dick

**Affiliations:** Department of Information Science, University of Otago, Dunedin, New Zealand

**Keywords:** Machine learning, Natural language processing, Word embedding, Class embedding, Humanitarian response

## Abstract

Humanitarian crises are unpredictable and complex environments, in which access to basic services and infrastructures is not adequately available. Computing in a humanitarian crisis environment is different from any other environment. In humanitarian environments the accessibility to electricity, internet, and qualified human resources is usually limited. Hence, advanced computing technologies in such an environment are hard to deploy and implement. Moreover, time and resources in those environments are also limited and devoted for life-saving activities, which makes computing technologies among the lowest priorities for those who operate there. In humanitarian crises, interests and preferences of decision-makers are driven by their original languages, cultures, education, religions, and political affiliations. Hence, decision-making in such environments is usually hard and slow because it solely depends on human capacity in absence of proper computing techniques. In this research, we are interested in overcoming the above challenges by involving machines in humanitarian response. This work proposes and evaluates a text classification and embedding technique to transform historical humanitarian records from human-oriented into a machine-oriented structure (in a vector space). This technique allows machines to extract humanitarian knowledge and use it to answer questions and classify documents. Having machines involved in those tasks helps decision-makers in speeding up humanitarian response, reducing its cost, saving lives, and easing human suffering.

**Supplementary Information:**

The online version contains supplementary material available at 10.1186/s41018-023-00135-4.

## Introduction

### Overview

Humanitarian response is a series of processes that aim at changing the state of crisis theatres from one of damage and destruction to one of relief and recovery. It is a complex operation of bringing life back to normality and easing the suffering of affected people. Treating patients, rescuing people, and saving lives are examples of what humanitarian response is about. Every process in humanitarian response produces large volumes of data that are captured, processed, used, and reused by humanitarian actors.

Humanitarian actors keep records on who responded to humanitarian crises, what response they provided, and where, when, why, and how they responded. They often save such information in a semi-standard data structure called the 6Ws, in which they preserve all aspects of their response in the form of questions and answers. The 6Ws data collection instrument and data exchange standard is used by humanitarian actors across the globe. It has been derived from the best practices used in education, journalism and law enforcement fields for survey and investigation (Eskildsen 2015; Hart 2017; Oxburgh et al. 2010; Stelfox 2009). A good story, a case study, or a police report is supposed to cover the six “big” W questions to capture every dimension of the story to be told.

However, answering such questions in a chaotic context such as the humanitarian environment is not an easy task. In sectors such as finance, agriculture, security, industry, and academia, reasonable computing infrastructures are usually a given, unlike in the humanitarian sector, where access to electricity, internet, and qualified human resources is usually limited. Hence, it is difficult to deploy and implement advanced computing technologies in this environment. Moreover, time and resources in humanitarian environments are also limited and devoted to life-saving activities, which makes computing technologies among the lowest priorities for those who operate there. In a humanitarian crisis, the interests and preferences of decision-makers are driven by their original languages, cultures, education, religions, and political affiliations. Therefore, imposing a universal solution upon them might be difficult, if not impossible.

The shortage in machine-based reasoning techniques that could be applied to humanitarian data to extract knowledge presents a real challenge. This challenge has been attracting the attention of the academic and humanitarian communities for years; and many attempts have been made to address it (Carsten and Chad 2015; Clark et al. 2015).

Currently, humanitarian knowledge is produced manually by domain experts. In this process, knowledge generation, resources are mostly spent on searching for information and reasoning to find answers. These two processes can be automated to save time and resources, improve quality, and reduce hazards. Having previously spent a lot of time finding information, human agents could then spend more time using human reasoning to extract knowledge from it.

### The problem

In humanitarian response to crises, thousands of questions are asked and answered daily. Most of the answers come from advisors, i.e. “domain experts”, who have knowledge and experience derived from previous humanitarian crises. The existing methodology of question answering relies on domain experts:

- Using their experience to transform existing information into knowledge, and
- Reasoning about extracted knowledge to answer humanitarian questions.

The existence and evolution of computing technologies in the past few decades has done very little to change the way questions are answered in humanitarian contexts. Human agents are still employed by organisations to answer questions in humanitarian crises in the absence of appropriate computing technologies. According to Fernandez-Luque and Imran (2018), Imran et al. (2020), Jomaa et al. (2016), Meier (2014), and Selanikio et al. (2002), this approach has many disadvantages:

1. The existing approach relies on domain experts who are usually hired as advisors, at the top of salary scales of humanitarian organisations, which makes it expensive to hire and retain them.
2. Computing techniques are rarely used to get answers. Getting answers is a human-dependent process, in which domain experts either read or remember the answers from what they experienced in the past, which makes it slow.
3. There is always limited time to provide answers (due to the urgent nature of humanitarian response). Hence, often the quality is sacrificed in favour of speed.
4. Domain experts are deployed to work in harsh and unsafe environments, where civil wars, diseases, hunger, and other harmful realities are present. Deploying human agents to do such work in every humanitarian environment is a dangerous mission as well.

Reducing the cost and time required to answer questions in humanitarian crisis could help humanitarian communities save more resources and time, which could be channelled towards the original mandate of these organisations, i.e. saving lives and reducing suffering.

### The proposal

We propose to embed humanitarian data in a continuous vector space, in which we place semantically similar concepts in close proximity to each other and then use geometric interpretation to infer knowledge from this vector space in order to augment the role of domain experts and enhance decision-making in humanitarian crises. To transform such knowledge into a vector space we may:

1. Uses a humanitarian text corpus to train a model on text classification based on a set of labels used to annotate observations. This text corpus has to be normalised to ensure that each document refers to a controlled set of labels. Using label annotations in text classification allows our model not only to retrieve known entities but also to predict and retrieve unknown entities based on the most plausible predictions.
2. Produces a neural text classification model through which questions can be answered, entities can be classified, analogies can be solved, and words can be embedded for humanitarian domain-specific contexts. This model should be able to transform unstructured plain text corpora into useful knowledge in a structured data format (with no human intervention).
3. Produces a humanitarian context-based word-embedding, in which related words can be placed closed to each other based on their humanitarian relations and context. For example, in this word embedding, the word “Japan” must be placed close to the word “Nuclear”, “JICA”, “Tsunami” and “Earthquake”, while in a generic word embedding “Japan” will be placed close to “Japanese”, “Korea”, “Tokyo”, and “China”. Having similar words placed in close proximity to each other allows knowledge retrieval at a later stage. In knowledge retrieval we use geometric distance, such as Cosine^1^ and Euclidean^2^ distance, to retrieve knowledge from the embedding, where the words that have relatively short distances between them are considered semantically related.
4. Given that we still need to have classes—and their affiliated words—placed in the right classes, we need to have a class embedding technique. Each entity in our model must belong to one of the classes. Our model uses a class embedding to connect classes and labels, associate them with corresponding texts, and optimise the distances between similar entities, classes, and properties to ensure that knowledge representation, in the model, matches the real humanitarian crisis environment. For example, if World Vision and Red Cross are close to each other in the real world (e.g. they have the same mandate, work in the same place, deal with the same population, and are funded by the same donor), they must be placed close to each other in our model’s vector space.

Using such a model we show that advanced computing techniques (such as machine learning) can be utilised to extract knowledge and insights from abandoned historical humanitarian records. This knowledge can be used by human agents in current and future crises to facilitate better, faster, and cheaper humanitarian response.

In the next section, we review and discuss related work, in which we explore three areas knowledge acquisition, knowledge representation, and knowledge extraction. In “Dataset description” section, we explain how we obtained, cleaned, processed, and prepared a humanitarian dataset that covers the period between 1994 and 2016 to be used in model training. In “The model” section, we present the mathematical model that we developed and used in this research and also illustrate the neural architecture of the model. In “Results visualisation” section, we infer and visualise the hidden semantic relations between humanitarian classes and documents. In “Evaluation” section, we evaluate the model: (1) qualitatively, though semantic measurement between humanitarian entities and document classification; and 2) quantitatively, through evaluation of the model accuracy across eight classes: agency, actor, SDG, sector, place, month, year, and reason using several techniques, including transformers. Finally, in “Conclusion” section, we conclude this paper and briefly discuss our results and achievements.

## Related work

Using advanced computing technologies, such as Machine Learning (ML) and Natural Language Processing (NLP), in the humanitarian sector has gained popularity in recent years. The reason for this popularity can be attributed to the desire of humanitarian organisations to speed up, reduce the cost, and improve the quality of their operations. Using advanced computing technologies, in such a process, helps in achieving these goals, as lessons learned from other sectors shows (Chou et al. 2010; Feng 2020; Fethi and Pasiouras 2010; Golding and Nicola 2019; González-Rivero et al. 2020; Mak and Pichika 2019; Mori et al. 2020; Tushar et al. 2018).

In this research, we focus on utilising such methods in improving knowledge inference and supporting faster and better decision-making processes, through which human suffering can be eased and reduced. We are interested here in finding prior research efforts, in the arena, to help us shape our research and avoid reinventing the wheel. In this section we review and discuss advanced computing technologies that are relevant to our research objectives.

In this section we study and explore two major areas of research of relevance to both humanitarian crises and advanced computing technologies. In particular, we are interested in: from where humanitarian data can be obtained (“Knowledge acquisition section”), how this data can be transformed into knowledge (“Knowledge representation” section), and how this knowledge can be used to improve humanitarian response (“Knowledge extraction” section).

### Knowledge acquisition

In humanitarian crises, acquiring reliable data is an essential task that humanitarian actors have to perform to enhance their decision-making stance. Sources of this data are varied, they might be collected from any viable source, including free text from social media, images from drones and satellites, and tabular data from APIs and websites. Most of the data sources, in humanitarian crises, are a mixture of different types, hence, different methodologies should be used with those data sources.

It is not enough to work on free text, using natural language processing to extract humanitarian knowledge while ignoring tabular data or aerial images, which hold a lot of humanitarian knowledge. Combining different data types together improves their quality and reliability. The quality of decisions made in humanitarian crises, relies—to a great extent—on the quality of the data used in making those decisions. In this section, we explore how data is acquired from different sources and how they are processed to produce reliable information that can be used by decision-makers to improve crises theatres.

Social media is an open source of humanitarian information. Social media platforms are often used by journalists, practitioners, and researchers to obtain timely information about ongoing crises. Imran et al. (2015) conducted a study to explore and review existing solutions that use information from social media to improve situation awareness, event detection and semantic enrichment during response to large-scale natural disasters. Similarly, Yin et al. (2012) used various data mining methods to classify, cluster, geotag, and visualise information captured from social media during natural disasters in order to enhance emergency situation awareness.

Besides social media, aerial data is another source of information in humanitarian crises. The deformation of terrains and damages of infrastructures in crisis theatres is a phenomenon that can be monitored and observed from open skies. Using aerial images to examine the impact of crises on earth is an approach that has been used for years to collect information about humanitarian crises. Ofli et al. (2016) proposed a framework in which they used combined human computing and machine learning to make sense of aerial data. In this framework, people are used to tag and label aerial images, while machine learning is used to learn and represent aerial images in a machine learning model that can be used to classify future images and help decision-makers taking better decisions.

Quinn et al. (2018) used machine learning and remote sensing data to map refugee settlements in crisis theatres in Africa and the Middle-East. They trained a machine learning model on satellite images collected from 13 refugee camps in South Sudan, Iraq, and Nigeria. They used this model to enumerate structures in those images and inform decision-makers in order to plan, estimate, and prepare their responses. To enrich their dataset, the authors used transferred learning from ResNet101 (He et al., 2015), which is a generic pre-trained image recognition model trained on the ImageNet^3^ dataset.

In some cases, data sources of different type are fused together to establish a multimodal data source. Ochoa and Comes (Ochoa & Comes, 2021) fused satellite images with tabular data to establish a machine learning workflow through which future disasters can be predicted and contained. They used data, of different types, from several sources: disaster characteristics from EM-DAT^4^, location-based risk from the World Bank^5^, INFORM index^6^, UN Data^7^, and satellite images from Google Maps. These datasets were pre-processed using different methods (feature extraction, dimensionality reduction, normalisation, and standardisation) and used to train the machine learning model. After training, the model was able to predict consequences of humanitarian crises, such as severity, deaths, casualties, affected people, and economic damages. Beside consequences, the model was also able to provide suggestions on possible solutions and what should be done in response to every disaster type.

The above-mentioned exercises used to be done manually, slowly, expensively, and inaccurately by humanitarian workers, where in the past humanitarian workers had to read tweets and blogs, line by line, to familiarise themselves with the new crises, read secondary data sources to understand the context in which the crisis took place, acquire and analyse aerial and satellite images to estimate damages on the ground, visit refugee camps and count the tents one by one to estimate the amount of required response. Using machine learning in the above examples saves enormous amounts of time for those who respond to humanitarian crises. This saved time can be better used to speed up the humanitarian response and reduce the suffering of those who are affected by the crises.

### Knowledge representation

Machine learning has many applications when it comes to humanitarian data representation. The work of Swamy et al. (2019) demonstrates the capability of machine learning to automate and replace the labour-intensive process of data labelling. In this work, they used 3659 tagged humanitarian datasets, obtained from the Humanitarian Data Exchange (HDX)^8^ to train a machine learning model for tag prediction. Datasets were tagged using the Humanitarian eXchange Language (HXL)^9^, which is a protocol for data exchange developed by Keßler and Hendrix (2015a) and widely adopted by humanitarian organisations to facilitate data interoperability. HXL aims at standardising the naming convention for the features in humanitarian datasets. For example, in some datasets users might use the word “camp” to refer to the place of the response while in other datasets the word might be “settlement”. In HXL, both location types might be hash-tagged as #camp. The model was later used to tag new, unseen, and untagged datasets. In this exercise the model achieved an accuracy of 94.3%.

Besides tabular data, plain humanitarian text can also be represented and embedded using machine learning methods. Unlike tabular data, in the previous example, textual data have no features, i.e. labels, to be learned. Hence, a few techniques were invented to deal with this matter, among which Word2Vec, GloVe, and FastText are prominent techniques with a remarkable success record. In this area, the work of Li et al. (2018) is particularly relevant to our research, where the authors trained their own word embedding using three famous word embedding techniques: Word2Vec, GloVe, and FastText. They trained their model on a humanitarian domain-specific text corpus harvested from Twitter. In this experiment they built a tweet classifier using supervised learning techniques. The aim of this classifier was to classify tweets as relevant or not relevant to humanitarian crises. They compared the different implementations of the model and found that GloVe produced a better result than the other two techniques.

Machine learning is not the only option when it comes to humanitarian data representation. There is a wealth of research on using other methods to perform this task, such as Semantic Web technologies, which have been used to represent humanitarian data over a long period. The work of Gaur et al. (Gaur et al., 2018) shows how Semantic Web ontologies are used to represent the concepts in humanitarian crises and reason about them to manage and plan humanitarian responses. The work of Limbu (2012) deals with vocabulary control issues, where in humanitarian crises people use different words to refer to the same concept. This work standardises this practice by providing a unified ontology, in which most of the vocabulary, used in humanisation crisis, is identified, retained, and used to standardise the language used in humanitarian crises. Later, Keßler and Hendrix (2015a) introduced a humanitarian ontology using HXL to facilitate data exchange in humanitarian crises. In this ontology, the authors created a taxonomy to classify humanitarian concepts and unify the language used to describe them. Using HXL helps humanitarian organisations to exchange their datasets at minimum cost using hashtags to label their dataset features.

### Knowledge extraction

Semantic similarity measurement is a common method used in knowledge extraction through which concepts of similar nature are enumerated, compared, and retrieved. It is one out of many measures used in this task. Besides semantic similarity, there are many other methods through which similar concepts can be retrieved, such as: semantic relatedness, semantic distance, taxonomic distance, semantic dissimilarity, and conceptual distance (Harispe et al. 2015). Among these techniques the most common is the semantic similarity, which is used in a wide spectrum of tasks in knowledge extraction.

There are two major methods to measure semantic similarity: corpus-based methods and knowledge-based methods, where the corpus-based methods are used to measure semantic similarity between words, documents, and meanings in textual, unstructured, data format (He and Lin 2016; Mandera et al. 2017), while the knowledge-based methods are used to measure semantic similarity in semi-structured data format such as graphs, ontologies, networks, and lexical resources (Harispe et al. 2015). Combining both methods together is also possible in hybrid semantic measurement methods.

Using semantic similarity in information retrieval has become increasingly popular over the past few years. In (Othman et al. 2017), word embeddings are used to embed historical questions and answers; and semantic similarity is used to retrieve answers for new questions that were never been asked before. The proposed method vectorises old questions and answers from online question answering archives, such as Yahoo! Answers, then vectorises new questions, measures the distance between new questions and old ones, and proposes answers to new questions based on their similarities to already-answered old questions.

In humanitarian crisis knowledge retrieval, there are multiple applications that cover a wide spectrum of humanitarian topics. For instance, knowledge retrieval in health crises can be used to detect disease outbreaks, such as COVID-19, where information about a disease can be obtained from public domain data sources and processed using machine learning methods and natural language processing to identify, screen, assess, and select relevant articles to a particular disease (Fernandez-Luque and Imran 2018).

Using machine learning in humanitarian knowledge retrieval is a well-known practice, in which social media platforms are monitored to retrieve, cluster, annotate, and rank up-to-date information about ongoing crises. In those applications, machine learning is used to classify social media content based on urgency, importance, severity, etc. Based on those classifications, the response of humanitarian decision-makers is planned and executed (Hürriyetoğlu et al. 2020).

Decision support systems can be used to retrieve knowledge from humanitarian data. For instance, decision-support systems can be used to prioritise resource allocation based on demand and actual needs of an affected population on the ground. Decision support systems provide adequate information to those who make decisions in humanitarian crises and allow them to take informed decisions on balanced resource allocation (Sahebjamnia et al. 2017). Balancing resource allocation saves lives and resources: (1) it saves lives because it allows those in dire need to have priority access to required resources, and (2) saves resources from being wasted and allocated to the wrong recipients who may not be in real need of them.

AI-enabled topic modelling can be used by humanitarian actors to retrieve useful knowledge in humanitarian context. The work of Imran et al. (2014) on Artificial Intelligence for Disaster Response (AIDR)^10^ applies a novel AI-enabled text classification and knowledge retrieval method to extract and retrieve the hidden knowledge in humanitarian data. They used AI to classify tweets into categories of interest to humanitarian decision-makers and allow them to be retrieved in real-time. AIDR has been developed and tested on a real crisis theatre (Pakistan Earthquake in 2013) to provide support to decision-makers. It achieved 80% accuracy on real-time data.

Besides advanced computing technologies that were discussed in this section, humanitarian actors have always the option to manually collect, process, and retrieve knowledge from humanitarian data repositories. However, we explained, earlier, in “The problem” section, why this human centred approach is not ideal. We identified four reasons for this approach to be exterminated: expensiveness, slowness, riskiness, and inaccuracy. We believe that the time has come for those obsolete manual methods to be modernised and augmented with new technologies that can do the same work in a faster, better, safer, and cheaper manner.

In the next section, we propose a machine learning model trained on historical humanitarian text corpora and generate new knowledge that can be employed to support decisions in humanitarian crises. In particular, we apply text embedding techniques to transform historical humanitarian text into a vector space to establish a semantically classified word embedding through which questions of decision-makers are answered.

## Dataset description

We used an annotated text corpus harvested from ReliefWeb through the Harvard Dataverse (Horwood 2017). Many records in the original dataset were missing labels. For example, a record about a certain crisis might have no reason given for an intervention or a country where that intervention took place. We ignored incomplete observations and only retained those observations which had at least one label per feature (as explained below). Therefore, out of 504,308 observations in this dataset, 52,808 observations were chosen, out of which 38,866 are used for training and 13,942 used to test and validate the model.

**Fig. 1:**
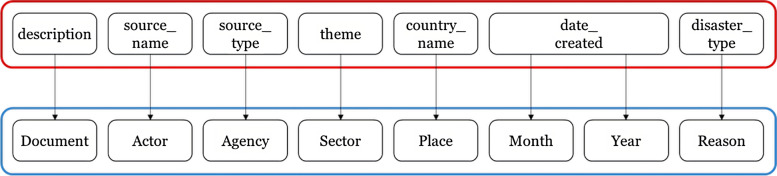
Mapping original dataset structure to new dataset structure

Besides observations, we had the following features in the dataset: description, source_name, source_type, theme, country_name, date_created, and disaster_type. While the first field is plain text, and the sixth field is a date, the rest of the fields have values from controlled vocabulary lists. Inspired by the 6Ws framework, we changed the names of these fields to match our naming convention in this research. Hence, source_name was changed to “actor”, source_type to “agency”, theme to “sector”, country_name to “place”, date_created to “year” and “month”, and finally disaster_type to “reason”. Figure 1 summarises this mapping exercise:

In this dataset, we have 1,028 actors, i.e. humanitarian organisations, assembled under seven broad categories, such as International Organization, Not-for-profit, and Government. We have also 19 humanitarian sectors, and 199 countries. The interventions, i.e. observations, in this dataset span 22 years of humanitarian operations from 1995 to 2016 (inclusive). We also have a label for each month in the year in which interventions took place. For each intervention, we have at least one reason out of 21 generic reasons for intervention, e.g. flood, earthquake, drought. At a later stage, we used semantic sentence encoding to enrich our dataset by adding 17 Sustainable Development Goals (SDGs).

Inspired by the work done in semantic document comparison, in particular the work of Carter et al. (2007), we developed a technique to detect the most similar SDG to each humanitarian intervention in our dataset based on Cosine distance between SDG vectors and vectors of observations in our dataset. We used a pre-trained sentence encoding model to vectorise observations in our dataset and descriptions of SDGs harvested from the United Nations official SDG website (United Nations 2020). We used these vector similarities to assign one SDG to each intervention in our dataset.

We also used text augmentation to amplify our dataset. Text augmentation is an NLP technique to amplify small text corpora, which is a common problem in domain-specific studies (Khabiri et al. 2019; Xu et al. 2018). Text augmentation can be done through similar and synonym word replacement, interpolation, extrapolation, random noise, word and sentence shuffling, syntax-tree manipulation, query expansion, semantic similarity augmentation, and round-trip translation (Giridhara et al. 2019; Marivate and Sefara 2019; Mnasri 2019). We used this technique to augment our dataset: we looked up words in each sentence, in every document, in our dataset, to find, and replace them with, their synonyms in WordNet. We repeated this process 10 times for every document in our dataset. Since we have 38,866 documents in our dataset, that increases the number of documents used to train our model to 388,660.

We found that our dataset is geographically imbalanced, where counties like India and Philippines have thousands of humanitarian crises in the past two decades compared to smaller, and more stable countries like New Zealand. There are many techniques used by research community to balance imbalanced datasets. There are two major techniques to balance imbalanced datasets, by either oversampling or under-sampling, where the former tend to synthetically inflate underrepresented classes, while the latter synthetically deflate overrepresented classes (Lin et al. 2017; Seiffert et al. 2008; Parsa et al. 2019). Both techniques were criticised, in many occasions, because they create synthetic data that do not represent the real imbalanced world when the representation of the real world is required as is (Seiffert et al. 2008).

While balancing the dataset is a prerequisite in many ML research projects, we found that balancing our dataset might yield wrong results. In general, balancing classes is argued as removing model bias towards more repetitive classes (He and Garcia 2009). However, in our case, this bias is a good feature to have; we want our model to be biased to places that are more exposed to humanitarian crises and to avoid treating every country in the world equally despite them being prone to crises or not. Hence, we decided addressing class imbalance was unwarranted.

A list of vocabulary has been extracted from the description (document) field in the dataset. The dictionary consisted of 145,833 unique words and phrases. We used the 10,000 most-used words out of these unique words and phrases.

## The model

### Mathematical model

Our mathematical model in this research is highly inspired by the work of Goldberg and Levy (2014) which is derived from the work of Mikolov et al. (2013a), particularly their Word2Vec Skip-Gram model, in which a Softmax function was used to model the probability of a context word (c) given a word (w) appearing nearby in the same sentence, with the embedding trained to maximise these probabilities for pairs of words appearing close in a corpus, and minimise them for other pairs. In this section, we will explain both works and how they influence our modelling technique.

The work of Mikolov et al. (2013a) used two techniques: Skip-Gram and Negative Sampling. While both concepts were successfully implemented together in Mikolov et al. (2013a), each has a different function and role that needs to be explained separately. On the one hand, the goal of Skip-Gram is to maximise the probability of the context words given a target word. In the original implementation of Word2Vec used Softmax function to maximise this probability as explained in Eq. (1).

1 $$p\left(w_O\left|w_I\right.\right)=\frac{\exp\left(\upsilon_{\mathit w\mathit o}^{\mathit'}\mathrm \top\upsilon_{\mathit w\mathit I}\right)}{\sum_{w=1}^W\exp\left(\upsilon_w^{'}\;\mathrm \top\upsilon_{wI}\right)}$$

where *w*_*O*_ is the context words and *w*_*I*_ is the target word, while ${\upsilon}_{w_O}^{\hbox{'}}$ and ${\upsilon}_{w_I}$ represent the vectors of context and target words, respectively. The implementation of Eq. (1) was computationally expensive given that the estimated number of pairs (*w*_*O*_, *w*_*I*_) is too large. Hence, a new method was proposed (Mikolov et al. 2013a), in which the Softmax function was replaced with a Hierarchical Softmax.

While the Hierarchical Softmax objective successfully addresses the computation cost problem, it was not able to address the noise problem, where logistic regression is expected to produce unintended noise (Mikolov et al. 2013a). Hence, a simplified version of the noise contrastive estimation (NCE) technique was introduced by Mikolov et al. (2013a), in which noise was eliminated through negative sampling.

For clarity, we present the formalisation of this approach by Goldberg and Levy (2014) who started by describing how to maximise the probability of the context words (c) given target words (w), where each word in Word2Vec is represented by a vector of weights, and (D) represents the set of the word/context pairs in the corpus, i.e. positive samples. On the other hand, in function (2) (Goldberg and Levy 2014), pairs of positive samples {target word, correct context word} and pairs of negative samples {target word, wrong context word} were used to maximise the probability of the positive samples, in (*D*), and minimise the probability of the negative samples, in (D^'^).

2 $$\arg\;\underset{\theta }{\max}\;\sum \limits_{\left(w,c\right)\in D}\log\;\sigma\;\left({\upsilon}_c\cdot {\upsilon}_w\right)+\sum \limits_{\left(w,c\right)\in {D}^{\hbox{'}}}\log\;\sigma \left(-{\upsilon}_c\cdot {\upsilon}_w\right)$$

According to Goldberg and Levy (2014), Skip-Gram with Negative Sampling (SGNS) is a technique used in the original Word2Vec implementation to “maximise the probabilities that all of the observations indeed came from the data [and] prevent all the vectors from having the same value, by disallowing some (w, c) combinations” ((Goldberg and Levy 2014), p. 3). In accordance with this definition, we created a derivative version of Goldberg and Levy’s (2014) probabilistic model, in which we used a derived version of Goldberg and Levy’s (2014) work on Negative Sampling, which has been explained earlier in Eq. (2), to create our own parametrised model that separates the embeddings of the documents and labels that have no co-occurrence in SEmHuS, as explained in objective (3).

3 $$\arg\;\underset{\theta }{\max}\;\sum \limits_{\left(d,L\right)\in T}\sum \limits_{l\in L}\log\;\sigma \left({\upsilon}_l\cdot {\upsilon}_d\right)+\sum \limits_{\left(d,L\right)\in {T}^{\hbox{'}}}\sum \limits_{l\in L}\log\;\sigma \left(-{\upsilon}_l\cdot {\upsilon}_d\right)$$

The difference between our work, in function (3), and the work of Goldberg and Levy (2014), in Eq. (2) is that in our loss function we optimise each document *d* with a set of labels *L*, i.e. one input document *d* to many output labels *L*, while in function (2) the optimisation is taking place between target word (w) and context word (c), i.e. one input word (w) to one output word (c). The loss function, in Eq. (3), optimises the correct tuples of document and labels (*d*, *L*) ∈ *T*, and penalises the wrong tuples (*d*, *L*) ∈ *T*^′^.

**Fig. 2:**
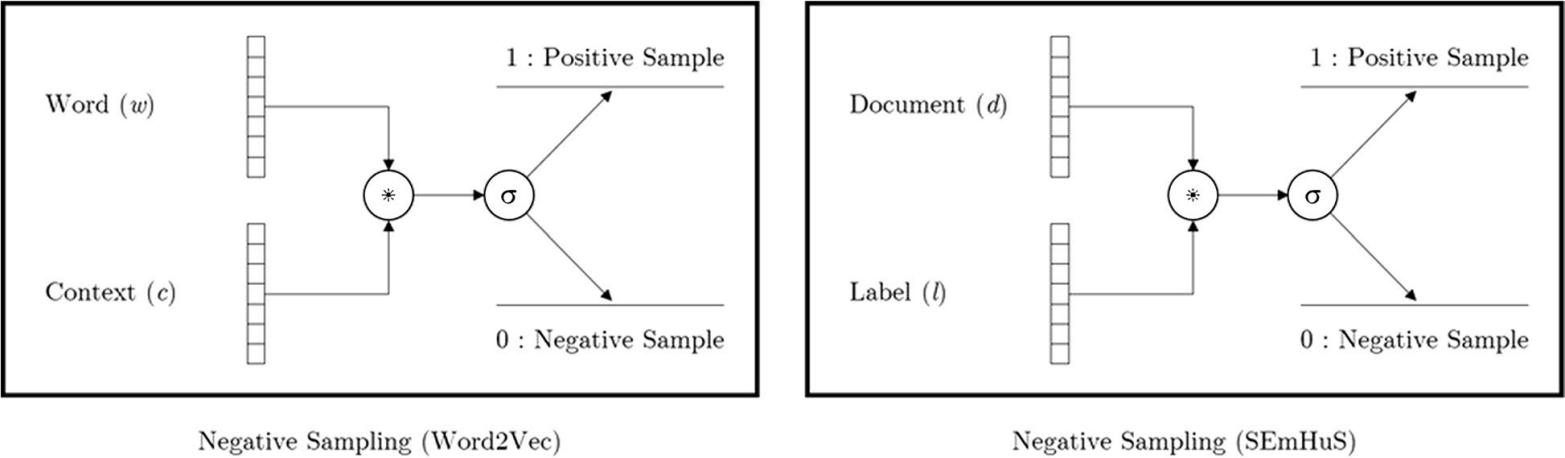
Negative Sampling in Word2Vec vs. SEmHuS

Figure 2 shows a comparison between the Word2Vec negative sampling technique and ours.

In our implementation of negative sampling:

1. We added a new binary label to our dataset, we call this label “State Label”,
2. Since observations in the dataset are correct, i.e. positive, their state label must be equal to one,
3. In every training epoch the model is learned on positive samples, but also generates a virtual batch of negative samples, i.e. wrong labels,
4. For negative samples, i.e. wrong labels, we made the value of state label equal to zero, and
5. We trained the model, and used a sigmoid function, to:
  - Activate positive samples (when the predicted State Label value is close to one) and,
  - Penalise negative samples (when the predicted State Label value is close to zero).

Using this technique, we managed to push away the wrong labels in the embedding and keep correct labels close to their corresponding documents.

### Neural architecture

SEmHuS was constructed to embed humanitarian data. A neural network based on Skip-Gram with Negative Sampling (SGNS) was designed to associate text inputs (documents) with tabular outputs (labels). The model was trained, validated, and evaluated using a corpus of 52,808 historical humanitarian observations. The model consists of one input layer, one embedding layer, and eight output layers:

1. The first layer is the Input Layer through which the vocabulary is passed into the network in batches of documents, each consisting of 100 words, in each training iteration. The total number of unique words in our text corpus is 145,833. However, for efficiency reasons, we used only the 10,000 most-used words (excluding stop words).
2. The second layer is the Embedding Layer, which maps each of the 10,000 inputs (one for each word in the dictionary, i.e. the vocabulary list) to a vector of 300 dimensions. The output of the Embedding Layer can be a sparse vector (where *each word* in the document is represented by a vector of 300 dimensions) or dense vector (where the *entire document* is represented by a vector of 300 dimensions) depending on the technique used to produce the document embedding^11^.
3. Next, we have eight Output Layers, one for each class, namely: Actor, Agency, SDG^12^, Sector, Place, Year, Month, and Reason Layer. The total number of labels in these output layers is 1325, and each label is represented by a one-hot encoded vector. Each output layer is activated using a Sigmoid function. The output from the Embedding Layer is used as the input for the Output Layers.

**Fig. 3:**
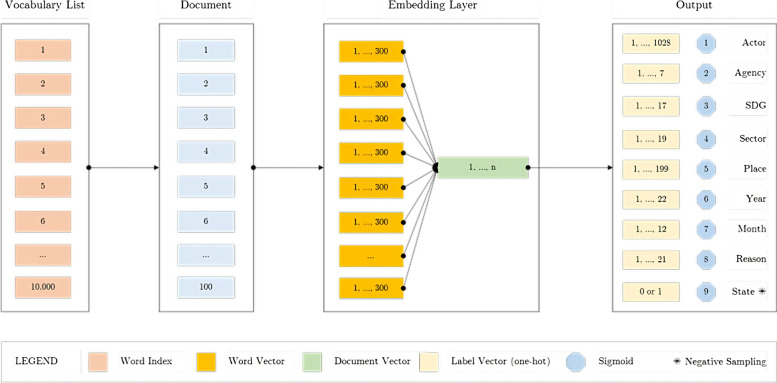
SEmHuS Model Architecture

An additional output (state label) was added to the model for Negative Sampling. This is a probability layer which has a binary output classification value of 1 or 0. It has been used to maximise the weights of positive samples (correct observations) and penalise the negative samples (wrong observations) (Fig. 3).

Each of the 10,000 words in our vocabulary list has been encoded into a unique index, and handed over to the Embedding Layer to convert them to vectors, which are either randomly initialised or obtained from pre-trained embeddings (more details in “Quantitative results” section). Those vectors are then passed to the output layers.

Each output layer in SEmHuS responds to one of the 6W questions: actor and agency layers respond to the question of “Who”, goal, i.e. SDG, and sector layers respond to “What”, place layer respond to “Where”, year and month layers respond to “When”, and reason layer respond to “Why”. The last question of “How”, i.e. subsector, has no specific label in the dataset, as it comes in the form of narrative text, which we used as an input layer in the model.

### Model anatomy

SEmHuS produced two embeddings: a document embedding and a class embedding. The first embedding is extracted from the weights *W* in the document embedding matrix, which come from the Input Layer (documents) towards the Embedding Layer *H*. The second embedding is extracted from the transposed weights ${V}_c^T$ going from the Embedding Layer *H* towards the Output Layer (labels).

**Fig. 4:**
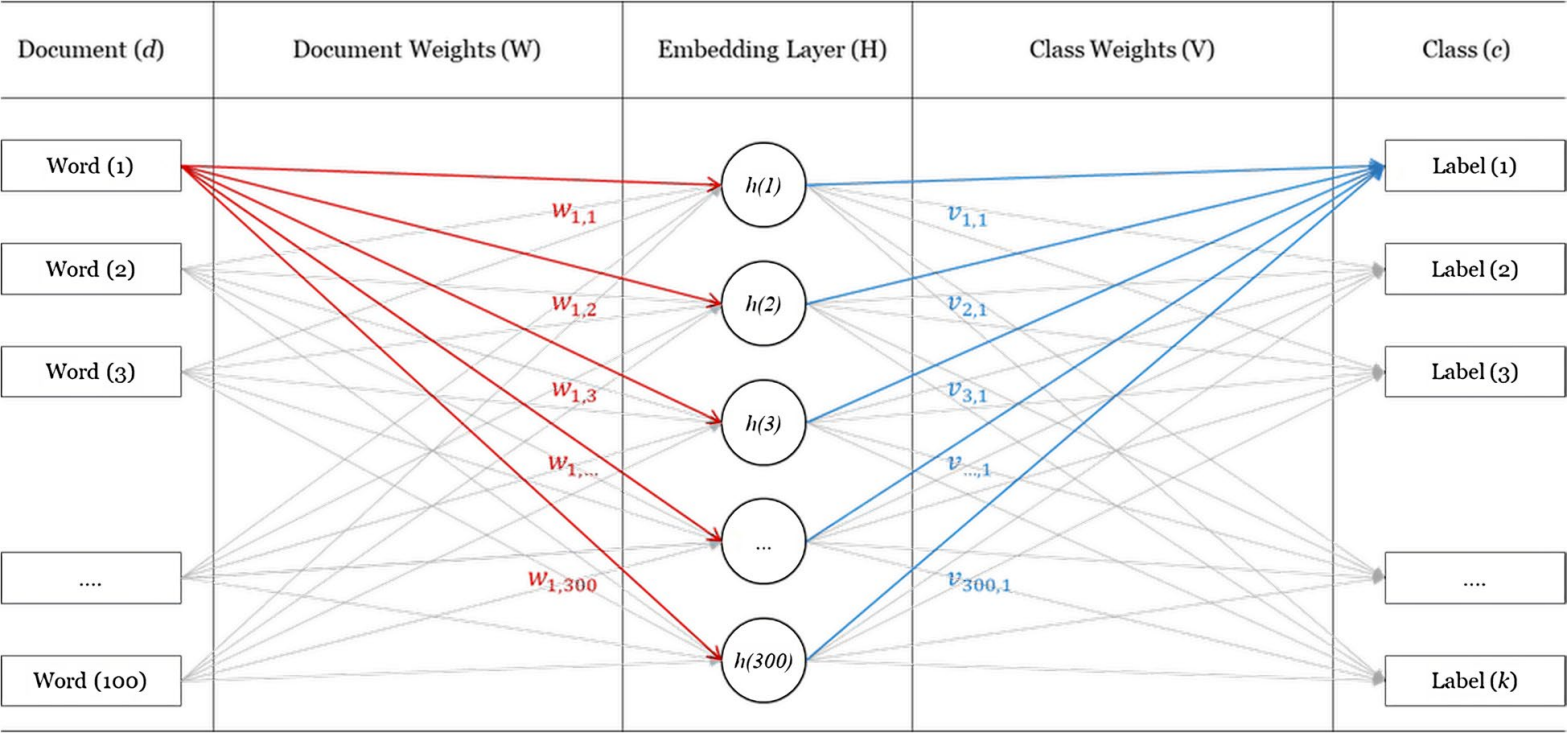
Text vs. class embeddings in SEmHuS

The embedding *W* holds the semantics of the documents used to train the model, where each word in this embedding has a vector of 300 dimensions. Meanwhile the transpose of the second embedding *V*_*c*_ holds the semantics of the labels, where each label in the model acquires a vector of 300 dimensions. Figure 4 shows how these two embeddings (document and class embeddings) are obtained:

The matrix ${V}_c^T$ consists of eight sub matrices (${V}_1^T$ to ${V}_8^T$), each of which responds to one of the output classes. The first embedding ${V}_1^T$ is a matrix of shape 7 × 300, where each humanitarian agency has a vector of 300 dimensions. The second ${V}_2^T$ is 1028 × 300 for actors, the third ${V}_3^T$ is 17×300 for SDGs, the fourth ${V}_4^T$ is 19 × 300 for sectors, the fifth ${V}_5^T$ is 199 × 300 for places, the sixth ${V}_6^T$ is 22 × 300 for year, the seventh ${V}_7^T$ is 12 × 300 for months, and finally the eighth ${V}_8^T$ is a matrix of 21 × 300 for reasons.

Embedding *V*_*c*_ implicitly records the semantics of the labels. It holds the relative distances between the embedding vectors of labels in eight classes: agency, actor, SDG, sector, place, year, month, and reason, providing semantic relations between those classes. Using this embedding to explore such relations helps in finding most similar entities in our model outputs. When two organisations, for example, operate in the same country, in the same year and month, doing the same or similar interventions, for the same reason, for the same SDG, that indicates they have a similar humanitarian history.

### Model potential

**Fig. 5:**
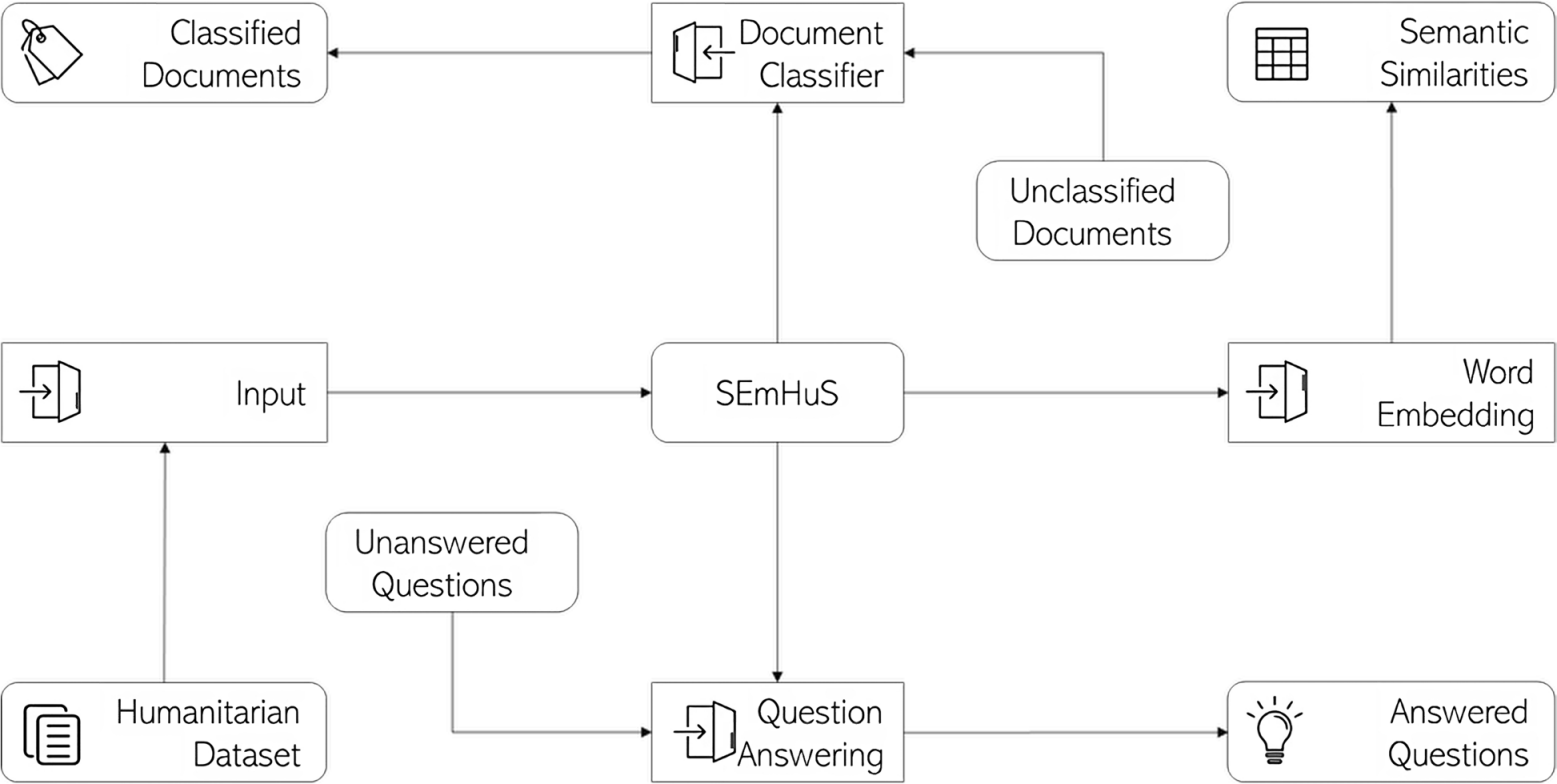
Model potential applications

SEmHuS consists of four components that have been developed to extract knowledge from historical humanitarian data. Using these components, the model can perform several tasks, such as answering questions, classifying documents, inferring knowledge, and measuring similarities. The following paragraphs describe the potential applications of SEmHuS and tasks that each application is able to perform:

- ❖ Class embedding: finds semantic similarity between entities used in records’ classes using a domain-specific, class embedding in which humanitarian labels are placed close to each other based on humanitarian history through which they passed together in the past. *This component is able to “infer knowledge” from humanitarian history.*
- ❖ Text embedding: finds semantic similarity between words used in records’ descriptions using a domain-specific, word embedding in which words are placed close to each other based on humanitarian relations between them. *Text embedding can measure semantic similarities, solve analogies, and discover unknown concepts.*
- ❖ Question answering: predicts results and extract knowledge from unstructured data sources. SEmHuS accepts questions from end users, as free text, and reasons about them to provide most plausible answers. *SEmHuS can “answer questions”.*
- ❖ Document Classification: transforms unstructured humanitarian data into a structured format. SEmHuS accepts free text as input and provides structured results as an output. *SEmHuS can “classify documents” and produce tabular results from plain texts* (Fig. 5)*.*

Out of the above-listed NLP tasks, associate with each potential application, we tested and evaluated SEmHuS against three of them in the next sections: measure semantic similarity (“Results visualisation” section), solve analogy (“Document embedding” section), and discover unknown concepts (“Class embedding” section).

## Results visualisation

This experiment was driven by our interest in discovering what our results look like in a two-dimensional space: what are the hidden relations between different documents in humanitarian contexts, and what can be revealed in a visual representation of results.

We used a t-SNE to transform output vectors into visualisations by reducing the dimensionality of the vectors (from 300 to 2 dimensions). t-SNE is a “technique for visualisation of similarity data that is capable of retaining the local structure of the data while also revealing some important global structure (such as clusters at multiple scales)” ((van der Maaten and Hinton 2008), p. 2599). In t-SNE, the similarity of the data is computed within a high dimensional space, and the visualisation projects vectors in that space into a two-dimensional one. Using t-SNE to visualise output victors is common and well-known technique, usually used in Computer Vision domain (Minhas 2021; Serebryakov 2020).

We transformed the output vectors into visualisations in three steps. In these steps, we

**Fig. 6:**
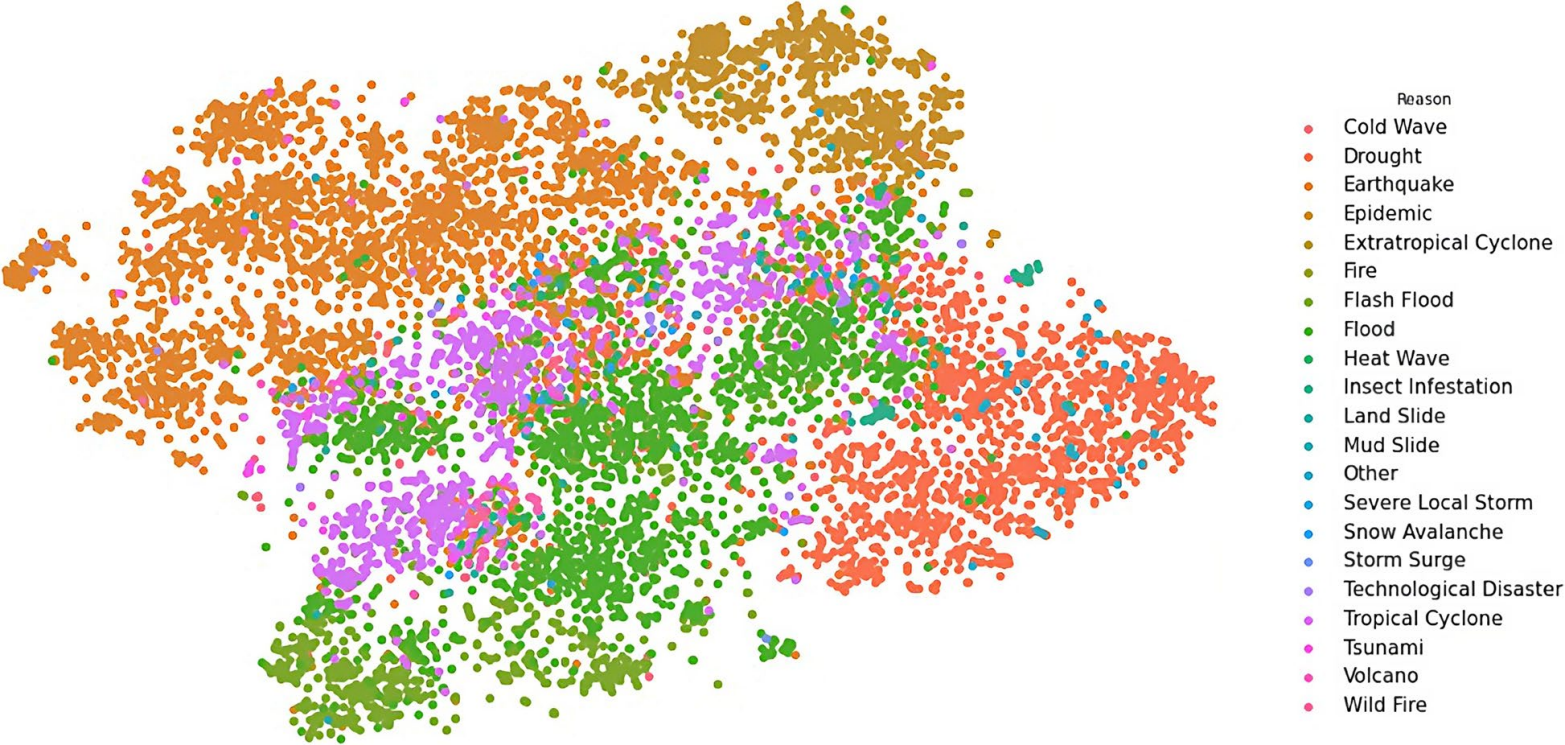
Visualisation of reasons of crises

**Fig. 7:**
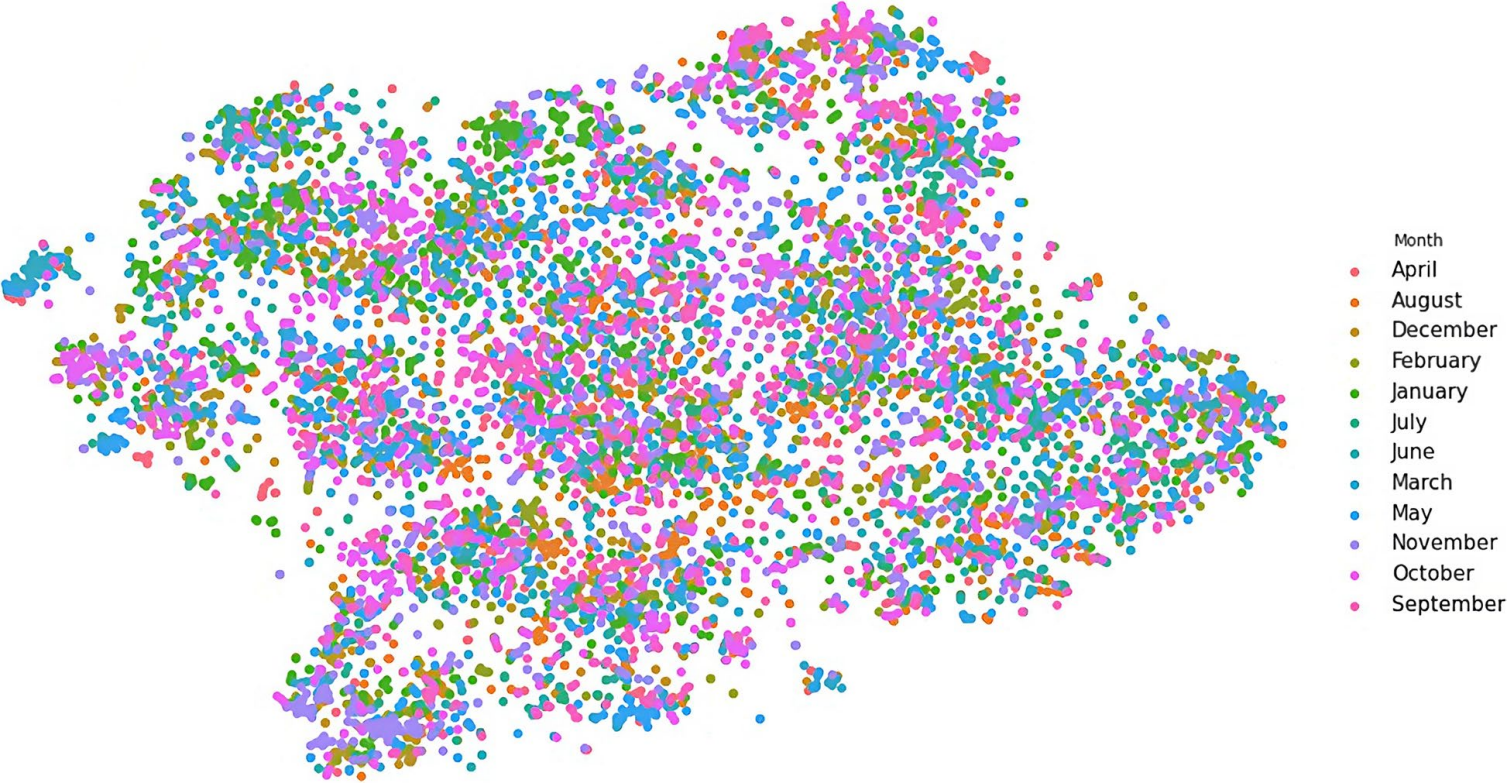
Visualisation of seasons of crises

**Fig. 8:**
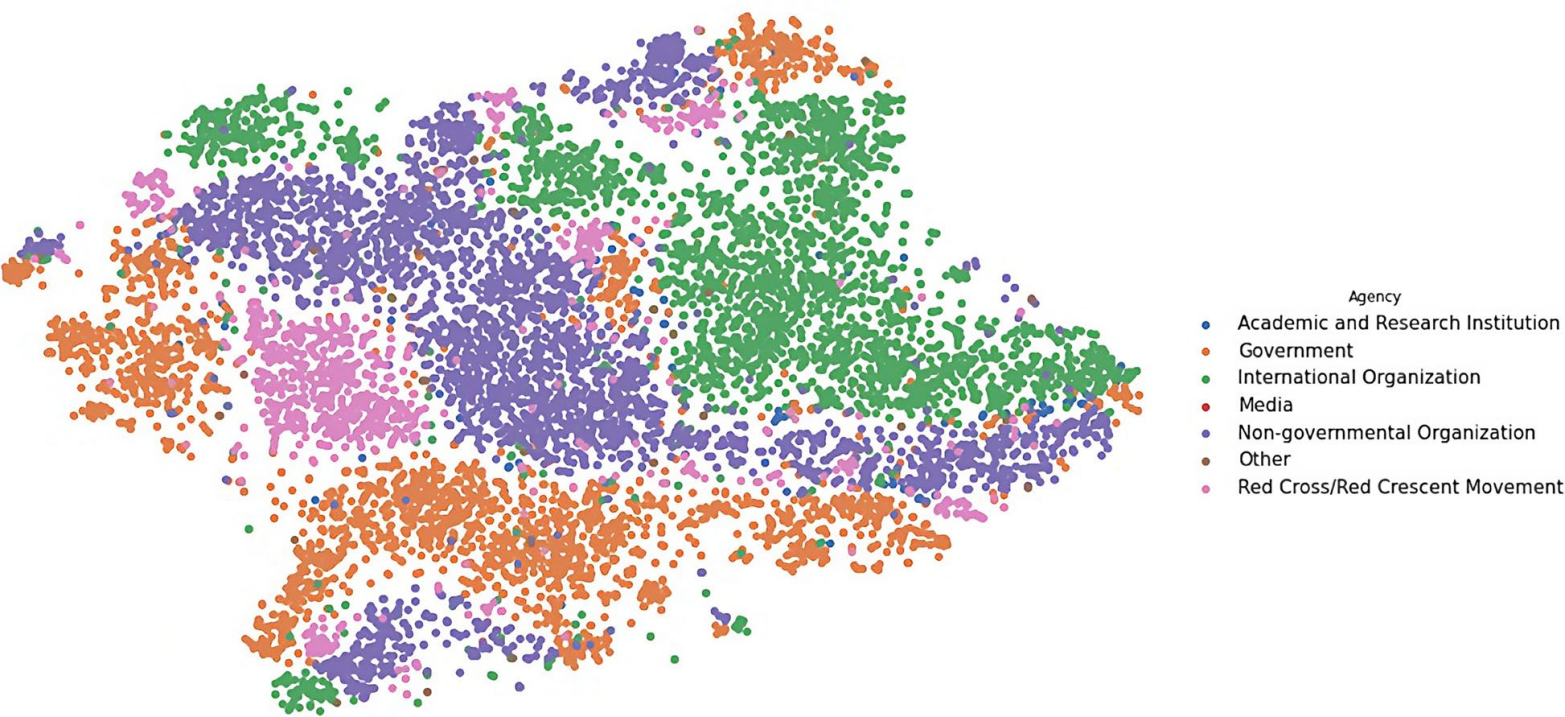
Visualisation of actors’ affiliation (agencies)

**Fig. 9:**
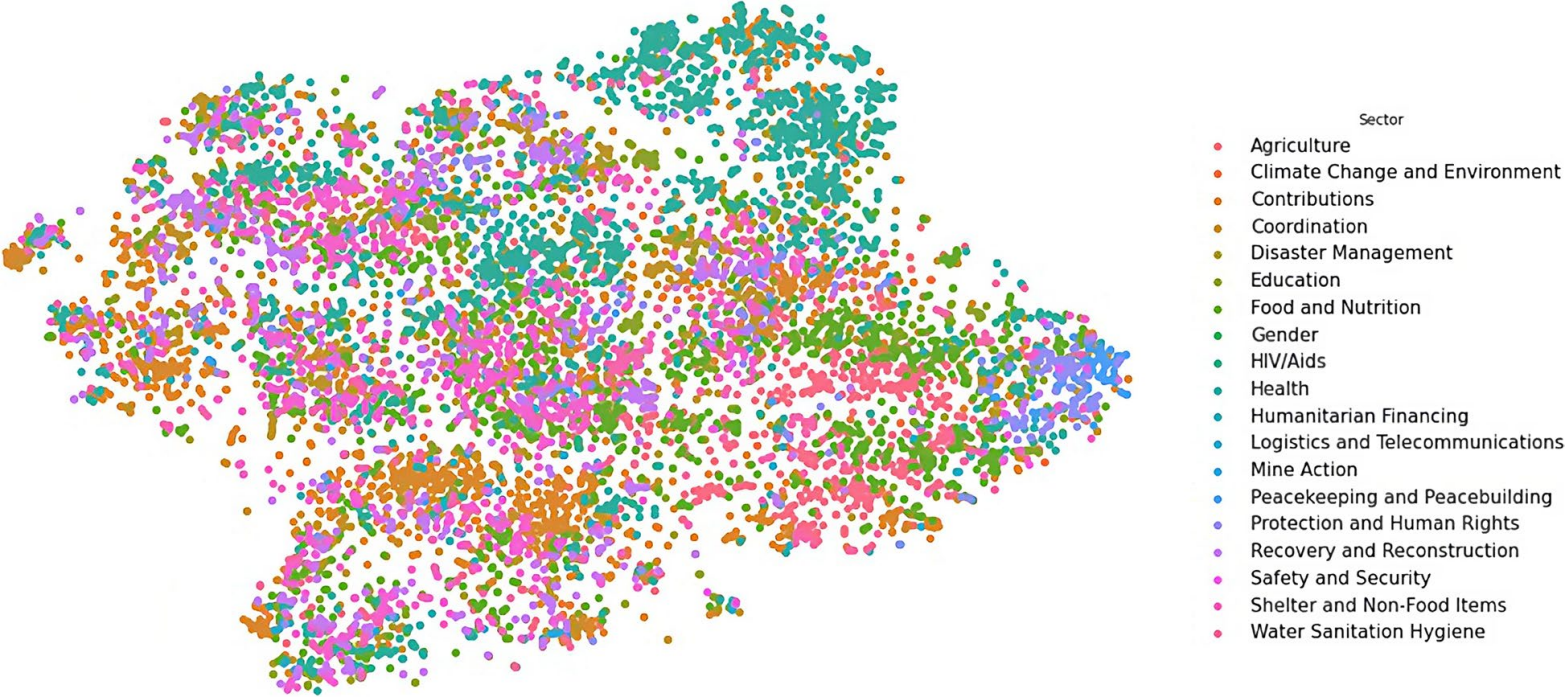
Visualisation of humanitarian sectors

**Fig. 10:**
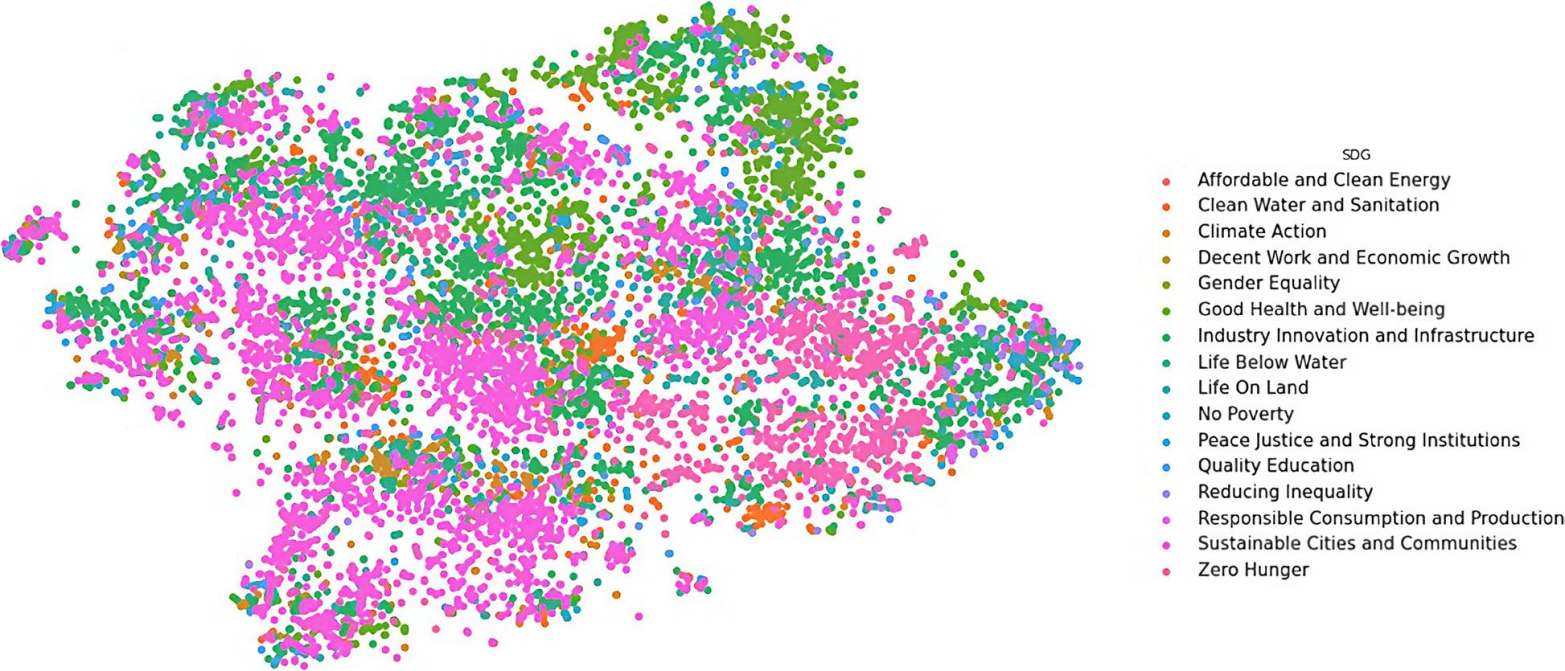
Visualisation of development goals

1. Concatenated the eight output vectors for each document we have in the dataset into one vector of 1325 dimensions for each document,
2. Reduced the dimensionality of the new vector from 1325 into two dimensions (X and Y) only, using the t-SNE technique (described in page 89), and then,
3. Plotted each observation on a two-dimensional canvas (using the Seaborn library in Python) as shown in Figs. 6, 7, 8, 9, and 10.

Dots, in the five visualisations, represent classified documents, where each document has eight classes: actor, sector, etc. Each visualisation represents one class and colours represent labels of the respective class. Colours, legends, and labels change from one visualisation to other while positions of the documents, i.e. dots, remain fixed.

The motivation to perform this task lies in four hypotheses we came to during this research: crisis seasonality, humanitarian mandates, humanitarian and development overlap, and cause and effect. Document visualisation provides a visual tool to evaluate these hypotheses, which will be explained and visualised in the next sections.

### Crisis seasonality

We hypothesised that similar crises take place in similar seasons. For instance, we presumed that tropical cyclones have seasons to take place in, while epidemics (such as dengue fever and flu) have different seasons. Hence, the visualisation of the reasons for crises should be close to the season of those crises.

While Fig. 6 shows that the reasons for crises are well clustered and separated from each other, Fig. 7 shows also that there is no noticeable correlation between reasons and seasons of crises for non-seasonal crises such as epidemic, earthquake, and volcano. These two figures show that such crises may take place at almost any time of the year. On the other hand, seasonal crises tend to cluster with seasons in which they are expected to take place. The best example of this phenomenon is the correlation between cold wave and winter, and heat wave and summer.

To further examine correlations, we transformed the above visualisations into a matrix, Additional file 1: Distance Correlation Matrix, Table A. 1. , in which columns represent months and rows represent reasons of crises. We measured the average distance between months and reasons and coloured those distances in green for short distances, red for long distances, and every other distance falls in a spectrum between these two colours.

Table A. 1. shows how cold months, such as January, February, and March have a stronger correlation to cold wave, while hot months such as June, July, and August have a shorter distance, i.e. stronger correlation, to heat waves. We can see also that there is no strong distance correlation between months and non-seasonal crises, such as volcano, tsunami, earthquake, and epidemic.

### Humanitarian mandates

On the other hand, the visualisation of the reasons (in Fig. 6) and the visualisation of the agencies (in Fig. 8) show some overlap between reasons of the crises and those who respond to those crises. We presumed that there will be overlaps between mandates of different agencies and reasons for response to crises, i.e. there is no specific agency in charge of a specific reason of crises. This presumption can be confirmed through the visualisation in Fig. 8.

Figure 6 shows that reasons of crises are clearly distinctive from each other, where each reason has a region on the map. The same for agencies (in Fig. 8), where each agency has its own territories. To make this correlation more visible, we measured the distances between agencies and reasons of crises. We calculated the average distances between agencies and reason of crises, and created a matrix, Additional file 1: Distance Correlation Matrix, Table A. 2, in which every column represents an agency and every row represent a reason of crisis. Then, we colour coded the distances from grey, for shorter distances, to white, for longer distances.

By comparing these two visualisations, using Table A. 2, we can see that government agencies, for instance, usually respond to the following reasons: extratropical cyclone, heat wave, and fire, while the mandate for non-governmental organisations spreads across fire, insect infestation, and technological disasters. Meanwhile media agencies are most interested in cold waves and earthquakes.

### Humanitarian and development overlap

The intersection between development and humanitarian domains is an active research area (Mechoulan et al. 2016). The international community has spent billions of dollars and decades of research on a quest to find and bridge the divide between these two domains (Blind 2019). In this research, we were motivated by the discussions in this area and the hope of discovering the missing links. Due to naming similarities between sector and goal names, we assumed functional similarities between them in humanitarian settings. For example, we expected the Food and Nutrition (sector) to overlay Zero Hunger (SDG). This assumption was not accurate, where we found that each sector extends over many goals and vice versa. We found also that some sectors are overlapping mainly with one development goal.

In Figs. 9 and 10 show how different humanitarian sectors overlap with development goals, where Fig. 10 shows, for example, how a development goal such as Good Health and Well-being in Fig. 10, overlaps with the Health Sector in Fig. 9. We can see also that some of the SDGs are overlapping with many sectors, such as in Zero Hunger, which spreads across the map, and mostly overlapping with Agriculture, Food and Nutrition, and Contributions sectors.

To make this comparison easier, we transformed the visualisations in Additional file 1 to a matrix, in which we measured the average distance between sectors and SDGs. In Additional file 1: Distance Correlation Matrix, Table A. 3, we coloured the shorter distances in green, longer ones in red, with every other distance coloured in a spectrum between red and green.

Table A. 3 shows that the nearest SDG to Education (sector) is Quality Education (SDG), to Water Sanitation Hygiene is Clean Water and Sanitation, to Food and Nutrition is Zero Hunger, to Gender is Gender Equality, to Health is Good Health and Well-being, etc. This table shows that our model discovered strong correlations between Sectors and SDGs. These correlations were not imposed on the model but rather discovered by the model in absence of human intervention.

### Cause and effect

We presumed that there is a correlation between reasons (Fig. 6) and sectors Fig. 9 in humanitarian crises. For example, the food (sector) must have a strong correlation with the drought (reason). However, we had no hard evidence of this presumption other than common sense and general knowledge. Therefore, we visualised both reason and sector results (in Figs. 6 and 9), to find out if this relation is valid. Both visualisations show that most of the reasons have no dedicated sector that responds to it, except for epidemic (reason) and health (sector), for which the visualisations (in Figs. 6 and 9) show a strong correlation between these two concepts. In the rest of the visualisation, we can see a mixture of sectors responding to each reason.

To make this correlation more visible, we measured the average distance between all entities in both classes, i.e. sector and reason, and below present them in a matrix that shows the average distance between each effect (Sector) and cause (reason). The matrix is in Additional file 1: Distance Correlation Matrix, Table A. 4, in which the green cells represent the shorter distances while the red ones represent the longer distances, and every other distance is in the spectrum between green and red.

In this table, we can see that the nearest reason to health sector is epidemic, to logistics is storm surge, to agriculture is insect infestation and drought, and to shelter and non-food items is cold wave and snow avalanche. These are just few examples among many in Table A. 4.

### Visualisation summary

The above visualisations show glimpses of the visual that SEmHuS enables. Many other combinations can be performed to show the overlap between different classes analysis in our model. By mixing different visualisations together, SEmHuS can produce up to 56 different comparisons of similarity to the above ones. Using those visualisations to present overlaps among actors, places, sectors, timeframes, and reasons provides a useful tool for the humanitarian community to use in supporting their day-to-day work.

## Evaluation

In this section, we used two evaluation methodologies to evaluate our proposed technique: quantitative and qualitative evaluations. While the former is a numerical comparison between results, the latter is more of a narrative and descriptive form.

In quantitative evaluation, we assess the quality of our results using several text embedding techniques. For qualitative evaluation we conducted two information retrieval tasks to assess the ability of our model to support and augment the role of domain experts in humanitarian crises.

### Quantitative results

As explained earlier, we transformed the documents in our dataset into vectors, which we used to train and optimise the model. There are several techniques to obtain those vectors; we used two prominent techniques that are used in similar tasks: (1) random initialisation, and (2) pre-trained vectors.

The first technique is a word average vector embedding technique, in which we create a vector for each word in our dataset, then randomly initialise those vectors, converge vectors through training, and consolidate them (either through concatenation or averaging) to create a unified vector for the document containing those words. Unlike the other techniques we evaluated, this technique does not borrow weights from pre-trained embeddings, but rather creates them from scratch. In the first model (AvgVec), the document embedding is obtained by averaging word vectors and using the obtained vectors to represent documents. Each document vector obtained from this technique is optimised to predict corresponding labels for each document. This embedding is generated using the Keras Embedding Layer. This technique is also used to obtain document vectors from other models, such as Bidirectional Encoder Representations from Transformers (BERT) (Devlin et al. 2019), A Lite BERT for Self-supervised Learning of Language Representations (ALBERT) (Lan et al. 2020), and Pre-training Text Encoders as Discriminators Rather Than Generators (ELECTRA) (Clark et al. 2019).

The second technique is a pre-trained document embedding technique, in which we assign a pre-optimised vector for each document. Pre-trained embeddings can be obtained from numerous sources and various techniques can be applied to create them. Examples of pre-training techniques used in similar NLP tasks, are Paragraph Vectors (Doc2Vec) (Dai et al. 2015), Neural Network Language Model (NNLM) (Iyyer et al. 2015), Universal Sentence Encoder (USE) (Cer et al. 2018). Table 1 shows the training results of our model using these techniques.

**Table 1:** Model accuracy using different embedding techniques

%	AvgVec	Doc2Vec	NNLM	USE	BERT	AL- BERT	ELEC- TRA
Actor	66.47%	72.61%	67.44%	72.67%	56.77%	50.96%	48.67%
Agency	92.65%	78.61%	69.35%	72.99%	94.78%	94.31%	95.43%
SDG	66.49%	58.61%	55.98%	57.38%	61.84%	59.93%	61.74%
Sector	55.70%	49.67%	48.97%	53.14%	55.19%	55.61%	56.66%
Place	79.41%	76.66%	72.32%	88.39%	84.70%	82.53%	73.95%
Year	74.01%	55.62%	34.73%	51.04%	83.81%	78.72%	80.81%
Month	63.84%	40.15%	22.93%	34.28%	70.41%	66.22%	69.62%
Reason	69.60%	57.97%	56.06%	61.11%	70.95%	71.31%	70.87%
Average	71.02%	61.24%	53.47%	61.37%	72.31%	69.95%	69.72%
Time	0H:58M	1H:12M	1H:24M	6H:28M	15H:18M	10H:47M	14H:43M

The first document embedding technique, Doc2Vec, is a shallow learning technique derived from the work of Le and Mikolov (2014). In this model, two techniques are fused to generate document vectors:

1. The first technique is paragraph vector with distributed memory (PV-DM) in which document vectors are acquired by training a model to predict target words in a document using context words plus paragraph ID then averages (or concatenate).
2. The second technique is paragraph vector with distributed bag of words (PVD-BOW), in which the document vector is obtained by training the model to predict all the words in a paragraph using paragraph ID.

The second document embedding technique, AvgVec, is also a shallow learning technique is, in which we use word vector averaging, where word vectors are randomly initialised and trained to obtain word vectors. By the end of the training process, we get a by-product word embedding.

Meanwhile, the third and fourth embedding techniques (NNLM and USE) both use a vector Deep Averaging Network (DAN). The difference between Doc2Vec, on the one hand, and NNLM and USE, on the other, is that Doc2Vec is a shallow learning technique (Le and Mikolov 2014), while NNLM and USE are deep learning techniques (Iyyer et al. 2015; Cer et al. 2018).

The last three techniques: BERT (Devlin et al. 2019), ALBERT (Lan et al. 2020), and ELECTRA (Clark et al. 2019) are transformer^13^ based techniques that use Masked Language Modelling (MLM), in which words are transformed into tokens, then in each training iteration the model replaces some of those tokens with [MASK] and optimise the model to predict those missing masked words.

ALBERT and ELECTRA are variations of BERT. ALBERT is a lighter version of BERT, evolved to reduce the extremely expensive computational cost of BERT. For comparison, while the internal structure of the base model of BERT has 108 million parameters, i.e. variables, ALBERT uses only 12 million (Lan et al. 2020).

Similar to the way in which BERT operates, ELECTRA masks some of the words in each sentence and optimises the model to predict those masked tokens. Unlike BERT, ELECTRA does not predict those masked words from the whole vocabulary list but rather from a fairly small list of plausible tokens produced by a generator network (Clark et al. 2019).

In this section, we tested and evaluated a number of text embedding techniques. We found that embedding performance is a compromise between resources, quality, and cost. It is widely assumed, by the research community, that the more computationally expensive models are slower to train, yet they yield better results; while the less computationally expensive models are faster to train, yet they yield poorer results in comparison to more expensive models. However, in our case, i.e. Table 1, we produced comparable results despite the size and cost of the models we used. In fact, some models break this rule and yield better results while being computationally inexpensive and fast to train. Example of those is the AvgVec technique. The reason for this good performance can be attributed to the amount of data we used in training process where more complex and computationally expensive models are designed to optimise large scale datasets unlike the dataset that we used in this research which has only 52,808 observations to optimise.

### Qualitative results

The model has been qualitatively evaluated in two informational retrieval tasks: class embedding and text embedding. In these two tasks, we used geometric distances to measure semantic similarities and ultimately use those similarities to retrieve results. Using geometric distance to measure semantic relations between words, documents, and concepts is a quite common information retrieval approach (Levy and Goldberg 2014a; Levy and Goldberg 2014b; Levy and Goldberg 2014c). It can be traced back to the early publications on text embedding between 1960s and 1990s (Borko and Bernick 1963; Doyle 1965; Dumais et al. 1988; Deerwester et al. 1990). In modern days, measuring semantic similarities through geometric distance can be found in many publications such as in (Goldberg and Levy 2014; Mikolov et al. 2013a; Mikolov et al. 2013b; Bojanowski et al. 2017; Thongtan and Phienthrakul 2019; Liao and Xu 2015; Kusner et al. 2015).

To retrieve results, we used Cosine and Euclidean distances. While the former is usually used in Natural Language Processing (NLP) tasks (Mikolov et al. 2013b; Mikolov et al. 2013a), the latter is more common in Computer Vision (CV) (Wang et al. 2005). However, the work of (Singh and Singh 2021; Qian et al. 2004; Xiong et al. 1970) shows that both Cosine and Euclidean distance can be used interchangeably in information retrieval tasks and both can produce comparable results. In the next sections, we will explain how the model performed in each one of these tasks, where Euclidean distance is used to retrieve documents in “Document embedding” section, while Cosine distance is used to retrieve classes in “Class embedding” section.

#### Document embedding

In this task, we used the *W* embedding from Fig. 4 to retrieve documents from our dataset using analogies. This task is inspired by the famous analogy in word embedding where “king is to man as queen is to woman” *vec*(Queen) ≈ *vec*(King) − *vec*(Man) + vec(Woman) (Allen and Hospedales 2019). In the past few years, this type of analogy has been widely investigated in word embedding research. It has been used and reused in numerous techniques to explain the geometric structure of words in a word embedding. This analogy belongs to a wider set of analogies, of a similar nature, such as Italy is to Rome as France is to Paris: *vec*(France) ≈ *vec*(Italy) − *vec*(Rome) + vec(Paris).

According to Allen and Hospedales (2019) these analogy queries on word embeddings work “surprisingly” well despite the fact that word embeddings are not trained to produce such a result but instead are trained using pairwise word co-occurrence to predict context words from target words or vice-versa. The justification for such a semantic relation is due to the hidden paraphrasing in such relations, where King paraphrases Royal and Man, while Queen paraphrases Royal and Woman. It follows that *vec*(Man) is to *vec*(King) as *vec*(Woman) is to *vec*(Queen).

In this task we used a set of analogies to retrieve documents by subtracting the vectors of unwanted terms, and adding vectors of new terms, to the document vector. For example, if we have a document, i.e. Doc 1, and this document is about “Ebola in West Africa”. We would like to find out what document will be the most similar to this document if we deducted the vector of the word “Ebola” and added the vector of the word “Cholera” to the document. We expect the result, of such substitution, to be a vector of a document Doc 2, about “Cholera in West Africa”, or a similar result, as shown in Eq. (4).

4 $$vec(Doc2)\approx \textrm{vec}(Doc1)- vec\left(\textrm{Ebola}\right)+ vec\left(\textrm{Cholera}\right)$$

To test this assumption, we randomly selected 10 documents from our dataset. We checked each one of them, manually, to decide what term to deduct and what term to add. We used our model to vectorise those documents and then used the NumPy library (in Python) to conduct the arithmetic operations, i.e. vector addition and deduction. We used the L2 Norm, i.e. Euclidean Distance, to measure the distances between *vec*(Doc 1) – *vec*(Unwanted Term) + *vec*(Wanted Term) on the one hand and *vec*(Doc2) on the other hand. In Table 2, we show the titles of the ten random (source) documents we chose and also show wanted and unwanted terms that we add and deduct.

**Table 2:** Source documents used in document retrieval task

No.	Source document (title)	Unwanted term	Wanted term
1	Malnutrition spikes in Rohingya communities	Flood	Earthquake
2	Anastasia Torres: Working for Her Community with the Hand of WFP in El Salvador	School	Hospital
3	Somalia meeting in Istanbul: 2.5 million people in dire need must not be forgotten	Cholera	Malaria
4	UNHCR rushes plastic sheeting and solar-powered lamps to Nepal earthquake survivors	Nepal	Syria
5	Press Conference on International Crisis Group’s Mali Report	Africa	Pacific
6	Responding to devastation: Update on EFICOR's tsunami relief intervention	Water	Food
7	IOM Helps 300 Haitian Migrants to Return Home Voluntarily from Dominican Republic	Refugees	Children
8	China: World Vision assesses relief needs in Henan	World Vision	Save the Children
9	New Zealand: PM announces Royal Commission on earthquake	Christchurch	Khartoum
10	Cultivating sustainable food security in Niger	Men	Women

Passing this set of documents, in Table 2, through the model, produces a set of ten target documents, which were expected to have preserved the semantics of the original source documents, minus the semantics of the unwanted terms, plus the semantics of the wanted terms. When we passed those documents to the model, we tasked the model to retrieve the target documents that has the shortest Euclidean distance to the original documents (after deducting the vectors of the unwanted terms and adding the vectors of the wanted terms to them). The results of this exercise are in Table 3.

**Table 3:** Target documents retrieved in document retrieval task

No.	Target document (title)	Euclidean distance
1	Indonesia: Aceh earthquake response situation report No. 17	5.6380
2	Pakistan: PRCS sets up hospital for victims in R. Pindi	4.1137
3	Australia commits $25 million to tackle malaria in the Pacific	5.2270
4	Refugees left out in cold in Middle East	7.2430
5	Marshalling the Pacific Response to the Climate Challenge	4.4651
6	United States of America Helps People Affected by Food Shortages in Malawi	2.8663
7	Improving Shelters for Child Victims of Trafficking in the Dominican Republic	3.4149
8	500,000 children affected by Thai floods	2.8555
9	Youth to the Rescue as Flooding Paralyzes Sudan	10.2920
10	UNMISS staff donate food to Kuajok flood victims	4.0483

Each target document, in Table 3, corresponds to a source document, in Table 2. The first column, in both tables, maps each target document to its corresponding source document. The Euclidean Distance, in the third column, shows the distance between each target document and its corresponding source document, where the shorter the Euclidean distance—between a source and target document—the stronger the semantic relation. Each target document (in Table 3) has the nearest vector to its corresponding source document (in Table 2) and consequently the new vector, i.e. *vec*(Source Document) – *vec*(Unwanted Term) + *vec*(Wanted Term), has the shortest Euclidean distance to the target document, i.e. *vec*(Source Document).

We notice, in Table 3, that distances between pair of documents are varied from pair to another, where some of those pairs have short distances while others have longer ones. These variations have no hidden meaning behind them. In the word embedding training process, words in the text corpus are assigned to randomly initiated vectors, then optimised by reducing the distance between these vectors based on the context in which the words are mentioned. When the words are mentioned in the same context, the distance between them is reduced. By the end of the training process, words in similar contexts are placed in close proximity to each other, i.e. the geometric distance between similar words is shorter than the distance between dissimilar words. The distance between similar words (and consequently documents) depends on the initial random values they were assigned to before training, as well as other training parameters, such as number of epochs, training duration, number of hidden layers, type of activation function, and size of the vectors.

We manually inspected the target documents to ensure that the model has retrieved reasonable results. In this inspection process we found a strong semantic relation between source documents and their corresponding target documents.

For instance, in the first result, we used the model to deduct the vector of the word “flood” and added the vector of the word “earthquake”, to the vector of first document, in Table 2. The model retrieved a document labelled “Indonesia: Aceh earthquake response situation report No. 17”, which is the first document in Table 3. The inspection shows that both source and target documents were discussing food and nutrition in the aftermath two different crisis in two different countries. We believe that our model retrieved the target document due to this similarity in the topic discussed in both of them.

Another example is in the fourth result, in which we replaced the vector of “Nepal” with the vector of “Syria”. While the source document was discussing UNHCR interventions in Nepal, the target document was about “Refugees left out in cold in Middle East”. The model found that when the vector of the word “Nepal” is deducted from the vector of a document discussing UNHCR work in Nepal, and then added the vector of “Syria” in its place, the resulted vector of the target document was so close to the original topic, i.e. refugees, which has been discussed in the source document.

The last example is the seventh result, in which we deducted the vector of the word “refugees” and added the vector of the word “children” to the source document. The inspection of this result shows that the source document was discussing refugees in the Dominican Republic, while the target document was about “Improving Shelters for Child Victims of Trafficking in the Dominican Republic”. In this example, we manually inspected the resulting, i.e. target, document to ensure that unwanted term is truly eliminated and replaced by the wanted term. While the source document^14^ is about helping Haitian refugees in the Dominican, the target document^15^ was about Haitian children in the Dominican Republic. We noticed that the model did not only push the vector of the document away from the vector of the word “refugees” but also brought the vector of the document close to the vector of the word “children”. Meanwhile, other terms, that were common in both documents, such as IOM, Return, Shelter, Home, Haiti, and Dominican remained in-place in both source and target documents.

#### Class embedding

In this task, we used the *V*_*c*_ embedding from Fig. 4 to qualitatively evaluate our model by finding entities similar to a random set of entities in the same class. The model has been tested to find places similar to Samoa, sectors similar to food and nutrition, years similar to the year 2015, etc.

Our model optimises the vectors of entities that have similar humanitarian co-occurrence, to lie in close proximity. To test and verify our model, we

- Generated a set of random entities, then extracted the vector of each one of those entities from matrix *V*_*c*_ (the transpose of the embedding layer),
- Measured the Cosine distance between the vectors of those entities to find the nearest neighbouring entities to each entity, and
- Ordered the retrieved entities from the nearest, i.e. shortest distance to the remotest, i.e. longest distance.

However, this approach of sorting entities in descending order based on their Cosine distance from the original entity, does not tell which result is more relevant and which one is less. It shows the relative relevance but it does not give a threshold for determining “sufficiently relevant” results to be returned to the user. To address this issue, we

- Measured the mean distance and standard deviation for all pairs of entities we have in our dataset,
- Used the mean and the standard deviation to compute a *z*-score, for every query, the *z*-score shows how many standard deviations are between the mean and the observed value (the distance),
- Set a threshold (*α*), i.e. a *z*-score value, where values (distances) lower than *α* are accepted and higher values are rejected, and
- Set the value of *α* at + 1.0. That means that we ignore the retrieved entities that have a distance longer than the mean distance + one standard deviation.

Table 4 shows the top results, i.e. the nearest neighbours, which their z-scores are less than or equal to *α* threshold.

**Table 4:** Neighbouring entities retrieved in entity retrieval task

Class	Entity	Neighbouring entities + *Z*-score (***α*** = 1.0)
Agency	International Organization	Non-governmental organization (0.7905); other (0.889);
Actor	New Zealand Red Cross	Tonga Red Cross Society (− 1.5067); Government of New Zealand (− 1.3277); Monaco Red Cross (− 1.3203); South African Red Cross Society (− 1.2625); Samoa Red Cross Society (− 1.1817); Australian Red Cross (− 1.1597); Fiji Red Cross Society (− 1.1321); ………………
SDG	Zero Hunger	Decent work and economic growth (− 0.1896); quality education (0.1785); peace justice and strong institutions (0.3656); no poverty (0.6263); life on land (0.8625);
Sector	Food and Nutrition	Agriculture (− 0.1187); water sanitation hygiene (0.8698); shelter and non-food items (0.8795);
Place	Samoa	Solomon Islands (− 3.7972); Cook Islands (− 3.6376); American Samoa (− 3.6284); Tonga (− 3.6136); Niue (New Zealand) (− 3.0536); Fiji (− 2.9669); Tuvalu (− 2.5095); Palau (-2.3772); Tokelau (− 2.3476); Marshall Islands (− 2.2438); ………………
Year	2010	2009 (− 0.5564); 2011 (− 0.3007); 2001 (0.5446); 2012 (0.6614);
Month	February	March (− 0.1951); April (0.2611); January (0.4965);
Reason	Earthquake	Tsunami (− 5.6487); technological disaster (0.2964); epidemic (0.5588); volcano (0.8883);

As shown in Table 4, the model, using the second embedding *V*_*c*_, yields meaningful results. The model autonomously discovered the following types of similarity: (1) geographical vicinity of the countries, (2) functional relations between organisations, (3) chronological order for timeframes, and (4) consequences of the disasters.

The model has never been informed that the Samoa is geographically close to Fiji and Tonga, that a tsunami is caused by an earthquake, February is close to March, April, and January, New Zealand Red Cross is similar to Australian Red Cross, or Zero Hunger (SDG 2) is most related to Decent Work (SDG 8). None of these meaningful relations were passed to—or imposed on—the model at any stage. They were discovered and captured by the model during the training process.

However, some of the similarities, in Table 4, might look odd from a non-humanitarian perspective, such as the similarity between food and nutrition and shelter and non-food items and the similarity between tsunami and technological disaster. Knowing that the non-food items include plates, pots, knifes, spoons, forks, cooking gas, stoves, and other kitchen appliances explains why these two sectors, i.e. food and non-food items, are close to each other.

Moreover, knowing that one of the most devastating tsunamis in recent history occurred in Japan in 2011, in the city of Fukushima, causing meltdown in three reactors of the city’s nuclear power plant, explains this unexpected similarity between tsunami and technological disaster.

This useful knowledge has been acquired by the model during the training process. These results show that *our model has developed its own sense of semantic connection* that makes it able to relate similar humanitarian entities to each other, without human intervention, and produce new knowledge out of the raw humanitarian records.

## Conclusion

In this paper, we developed a machine learning model through which the divide between historical humanitarian crises and existing ones is bridged. We built a model that translates uncontrolled vocabulary and unstructured humanitarian text into classified structures. We transformed a dataset of documents and classes into a vector space, in which semantic similarities between humanitarian entities were established, measured, and used to improve our understanding of the interactions of humanitarian entities in humanitarian environments. A unique approach has been established to extract meaningful results from historical humanitarian records. Out of six NLP tasks, listed in “Model potential” section, we tested SEmHuS against three of them (measure semantic similarity, solve analogy, and discover unknow concepts) and achieved meaningful results. Moreover, the embeddings visualisation, in “Results visualisation” section, revealed some hidden relations between humanitarian concepts, such as crisis seasonality, humanitarian mandates, humanitarian and development overlap, and cause and effect of humanitarian crises. We managed to bring this knowledge to the light and use it to understand the underlaying semantic relations between humanitarian concepts. Those relations used to be buried—in the past—under piles of historical humanitarian records. This revelation helps in making future humanitarian responses faster, cheaper, safer, and more efficient.

## Supplementary Information

**Additional file 1: **Distance correlation matrix. **Table A. 1.** Crises seasonality distance correlation. **Table A. 2.** Humanitarian mandates distance correlation. **Table A. 3.** Humanitarian and development distance correlation. **Table A. 4.** Cause and effect distance correlation.

## Data Availability

Data and source code are available at https://github.com/shamoug/SEmHuS.

## Footnotes

1 https://reference.wolfram.com/language/ref/CosineDistance.html

2 https://reference.wolfram.com/language/ref/EuclideanDistance.html

3 ImageNet is a large database of images initiated in 2006 by a group of Computer Vision researchers at the University of Princeton. Many papers were published about ImageNet. The first published paper can be found in (Deng et al. 2009). The web address of ImageNet is https://www.image-net.org/

4 Emergency Events Database (EM-DAT) is one of the largest and most comprehensive databases on natural and technological disasters. It has been established in 1988 by Centre for Research on the Epidemiology of Disasters (CRED) at the University of Louvain through an initial funding from World Health Organization (WHO). EM-DAT keeps records of all disasters from 1900 till today, i.e. 2022. The web address of EM-DAT is https://www.emdat.be/

5 Open Knowledge Portal of the World Bank is the World Bank's official open access repository for World Bank research outputs and knowledge products. The web address of Open Knowledge portal is https://openknowledge.worldbank.org/

6 INFORM Index is a project funded by European Union to assess and profile countries based on their vulnerability to crises. The web address of INFORM Index is https://drmkc.jrc.ec.europa.eu/inform-index

7 UN Data is the official United Nations statistical data portal. It provides official statistics of the UN since 2008. The web address of UN Data is https://data.un.org/

8 The Humanitarian Data Exchange (HDX) is an open access web portal established by the United Nations (OCHA) in 2014 to host and share data across humanitarian crises. The web address of HDX is https://data.humdata.org/

9 The Humanitarian eXchange Language (HXL) is project by the United Nations (OCHA) that aims at “refining data management and exchange for disaster response” (Keßler and Hendrix 2015a). The web address of HXL is https://hxlstandard.org/

10 The Artificial Intelligence for Digital Response (AIDR) is an open access web platform. It uses AI/ML to process and classify crisis related social media messages. Established and launched in 2015 by Qatar Computing Research Institute (QCRI). The web address of AIDR is http://aidr.qcri.org/

11 For example, if the document size is 100 words, the sparse vector will be a concatenation of word vectors that makes the embedding size of the document equal to 300 dimensions (per word) by 100 words (per document), which produce a document vector of 30,000 dimensions. The other option is to average the word vectors and produce a dense document vector. In this option the document embedding will be 30,000 dimensions divided by number of words in the document. The result will be a dense vector of 300 dimensions that represent the entire document.

12 Sustainable Development Goals https://sdgs.un.org/goals

13 Transformer is a full-attentional convolutional feed-forward neural network architecture, which emerged from Recurrent Neural Network (RNN), and is used in sequence modelling tasks to capture word-level sequential context (Dehghani et al. 2019; Wang et al. 2019).

14 https://reliefweb.int/report/haiti/iom-helps-300-haitian-migrants-return-home-voluntarily-dominican-republic

15 https://www.iom.int/news/improving-shelters-child-victims-trafficking-dominican-republic
