# Supplementary material for: SEmHuS: a semantically embedded humanitarian space
Source: J Int Humanit Action. 2023 Mar 7;8(1):3. doi: 10.1186/s41018-023-00135-4 (PMC9990040; doi:10.1186/s41018-023-00135-4)
Supplement: Supplementary file 1 — Additional file 1: Distance correlation matrix. Table A. 1. Crises seasonality distance correlation. Table A. 2. Humanitarian mandates distance correlation. Table A. 3. Humanitarian and development distance correlation. Table A. 4. Cause and effect distance correlation. [file 41018_2023_135_MOESM1_ESM.docx]

Additional file

Additional 1. Distance correlation matrix

Table A. 1. Crises seasonality distance correlation

| Month  reason | 1 | 2 | 3 | 4 | 5 | 6 | 7 | 8 | 9 | 10 | 11 | 12 |
| --- | --- | --- | --- | --- | --- | --- | --- | --- | --- | --- | --- | --- |
| Cold wave | 0.849 | 0.858 | 0.865 | 0.984 | 1.072 | 1.076 | 0.951 | 1.075 | 0.923 | 1.084 | 0.990 | 1.033 |
| Drought | 0.997 | 0.980 | 1.026 | 1.016 | 1.018 | 0.974 | 0.959 | 0.965 | 0.984 | 1.029 | 0.993 | 1.059 |
| Earthquake | 0.915 | 0.981 | 0.972 | 0.948 | 0.962 | 0.987 | 1.055 | 1.039 | 1.058 | 0.999 | 1.056 | 1.052 |
| Epidemic | 0.981 | 0.979 | 1.010 | 1.034 | 0.992 | 1.004 | 0.997 | 0.979 | 0.971 | 0.982 | 0.984 | 1.039 |
| Extratropical cyclone | 0.999 | 0.974 | 1.093 | 1.035 | 0.968 | 1.050 | 0.959 | 1.028 | 0.955 | 0.953 | 1.014 | 1.006 |
| Fire | 1.069 | 0.993 | 1.007 | 0.944 | 1.056 | 1.004 | 1.036 | 0.794 | 1.038 | 1.026 | 1.057 | 1.029 |
| Flash flood | 1.133 | 1.059 | 1.039 | 1.029 | 1.008 | 0.978 | 0.969 | 0.860 | 1.004 | 0.962 | 0.945 | 0.900 |
| Flood | 1.018 | 1.020 | 1.058 | 1.001 | 0.974 | 1.024 | 0.980 | 0.938 | 0.978 | 0.994 | 0.988 | 1.036 |
| Heat wave | 1.044 | 1.019 | 1.066 | 1.046 | 1.017 | 0.912 | 0.900 | 0.881 | 1.005 | 1.099 | 1.040 | 1.088 |
| Insect infestation | 1.076 | 1.006 | 1.047 | 0.965 | 1.024 | 0.987 | 0.893 | 0.860 | 1.002 | 1.002 | 0.980 | 0.976 |
| Land slide | 1.062 | 1.006 | 1.004 | 1.013 | 0.990 | 0.986 | 1.046 | 0.971 | 0.932 | 0.972 | 0.956 | 0.927 |
| Mud slide | 1.141 | 1.119 | 1.048 | 1.033 | 0.878 | 0.931 | 1.011 | 0.946 | 0.912 | 0.842 | 0.990 | 0.982 |
| Other | 1.034 | 1.025 | 1.020 | 0.966 | 0.982 | 0.957 | 0.909 | 0.992 | 0.978 | 1.035 | 1.014 | 1.037 |
| Severe local storm | 0.982 | 0.891 | 0.892 | 1.015 | 0.934 | 0.916 | 0.981 | 1.104 | 0.988 | 1.042 | 1.136 | 0.968 |
| Snow avalanche | 0.911 | 0.831 | 0.916 | 0.897 | 0.961 | 1.003 | 1.057 | 1.050 | 1.081 | 1.059 | 1.037 | 1.000 |
| Storm surge | 0.963 | 0.975 | 0.843 | 0.938 | 0.990 | 0.986 | 1.073 | 1.074 | 1.140 | 1.051 | 0.817 | 0.922 |
| Technological disaster | 1.068 | 1.087 | 0.864 | 0.962 | 0.926 | 0.948 | 0.947 | 0.996 | 0.988 | 1.071 | 1.007 | 1.001 |
| Tropical cyclone | 0.955 | 1.014 | 0.994 | 0.991 | 1.031 | 1.094 | 1.047 | 1.062 | 1.026 | 0.932 | 0.928 | 0.923 |
| Tsunami | 0.922 | 0.965 | 0.934 | 0.938 | 0.979 | 1.030 | 1.052 | 1.108 | 1.049 | 1.019 | 1.061 | 0.901 |
| Volcano | 0.954 | 0.997 | 1.071 | 1.009 | 1.041 | 0.986 | 0.988 | 1.009 | 1.031 | 0.929 | 0.960 | 0.983 |
| Wild fire | 1.062 | 0.927 | 1.096 | 0.955 | 1.018 | 1.051 | 1.005 | 0.891 | 0.978 | 1.067 | 1.002 | 0.940 |

Table A. 2. Humanitarian mandates distance correlation

| Agency  reason | Academic and research institution | Government | International organization | Media | Non-governmental organization | Other | Red Cross/Red Crescent Movement |
| --- | --- | --- | --- | --- | --- | --- | --- |
| Cold wave | 1.015 | 1.034 | 1.005 | 0.814 | 1.058 | 0.954 | 0.967 |
| Drought | 0.995 | 1.011 | 1.002 | 1.006 | 1.016 | 0.932 | 1.053 |
| Earthquake | 1.031 | 1.033 | 1.037 | 0.872 | 0.995 | 0.962 | 0.986 |
| Epidemic | 1.017 | 0.998 | 0.982 | 0.967 | 1.050 | 0.979 | 1.030 |
| Extratropical cyclone | 1.091 | 0.896 | 0.943 | 1.002 | 1.002 | 0.959 | 0.935 |
| Fire | 1.010 | 0.955 | 0.893 | 0.935 | 0.983 | 1.066 | 0.996 |
| Flash flood | 1.035 | 1.025 | 1.012 | 1.064 | 1.007 | 1.030 | 1.003 |
| Flood | 1.001 | 0.995 | 0.978 | 1.000 | 1.009 | 1.004 | 1.003 |
| Heat wave | 1.000 | 0.914 | 0.936 | 1.026 | 1.052 | 0.949 | 0.967 |
| Insect infestation | 0.998 | 1.000 | 0.939 | 1.043 | 0.983 | 1.050 | 1.030 |
| Land slide | 0.931 | 1.016 | 1.070 | 1.000 | 1.020 | 0.972 | 1.016 |
| Mud slide | 0.922 | 1.055 | 1.026 | 1.021 | 0.993 | 1.033 | 0.988 |
| Other | 0.966 | 1.045 | 1.018 | 1.025 | 1.008 | 0.877 | 1.039 |
| Severe local storm | 1.126 | 0.947 | 0.946 | 0.898 | 0.995 | 1.048 | 0.909 |
| Snow avalanche | 0.964 | 0.958 | 0.989 | 0.964 | 1.015 | 1.013 | 1.043 |
| Storm surge | 0.964 | 1.049 | 1.058 | 0.946 | 1.034 | 0.967 | 1.045 |
| Technological disaster | 0.970 | 1.005 | 1.009 | 0.993 | 0.989 | 1.062 | 0.972 |
| Tropical cyclone | 0.936 | 1.010 | 1.026 | 1.024 | 1.041 | 1.028 | 0.994 |
| Tsunami | 0.995 | 1.006 | 1.023 | 0.966 | 0.993 | 1.026 | 0.991 |
| Volcano | 1.074 | 1.010 | 0.978 | 0.999 | 1.050 | 1.026 | 0.999 |
| Wild fire | 0.922 | 0.985 | 0.986 | 0.930 | 1.055 | 0.986 | 0.973 |

Table A. 3. Humanitarian and development distance correlation

| SDG  sector | Affordable and clean energy | Clean water and sanitation | Climate action | Decent work and economic growth | Gender equality | Good health and well-being | Industry innovation and infrastructure | Life below water | Life on land | No poverty | Peace justice and strong institutions | Quality education | Reducing inequality | Responsible consumption and production | Sustainable cities and communities | Zero Hunger |
| --- | --- | --- | --- | --- | --- | --- | --- | --- | --- | --- | --- | --- | --- | --- | --- | --- |
| Agriculture | 1.062 | 0.945 | 1.039 | 1.023 | 0.938 | 1.098 | 0.943 | 1.029 | 0.866 | 1.075 | 1.125 | 1.061 | 0.946 | 0.973 | 1.037 | 0.767 |
| Climate change and environment | 0.894 | 0.961 | 0.880 | 1.100 | 0.864 | 1.097 | 0.955 | 0.900 | 0.839 | 1.087 | 0.976 | 1.052 | 1.073 | 0.676 | 1.128 | 0.971 |
| Contributions | 0.962 | 1.026 | 0.901 | 0.982 | 1.094 | 0.958 | 0.985 | 1.020 | 1.091 | 0.891 | 0.919 | 0.953 | 0.875 | 1.059 | 1.020 | 0.998 |
| Coordination | 0.996 | 0.946 | 0.932 | 0.998 | 1.123 | 1.017 | 0.976 | 0.937 | 0.933 | 1.083 | 0.974 | 1.054 | 0.997 | 0.995 | 0.967 | 1.029 |
| Disaster management | 0.907 | 1.058 | 0.838 | 1.044 | 1.034 | 0.974 | 0.930 | 1.019 | 0.894 | 1.036 | 1.118 | 0.985 | 1.131 | 0.826 | 0.950 | 1.014 |
| Education | 0.967 | 1.022 | 1.006 | 0.869 | 1.077 | 1.059 | 0.972 | 1.006 | 0.986 | 0.982 | 1.068 | 0.639 | 0.957 | 1.011 | 1.020 | 0.918 |
| Food and nutrition | 1.061 | 1.011 | 1.068 | 0.980 | 1.003 | 0.974 | 1.007 | 1.080 | 1.028 | 0.924 | 1.018 | 1.062 | 1.052 | 0.977 | 1.021 | 0.772 |
| Gender | 0.933 | 0.989 | 1.071 | 0.808 | 0.726 | 1.001 | 1.010 | 1.031 | 0.945 | 0.930 | 1.020 | 0.934 | 0.985 | 0.956 | 1.073 | 0.976 |
| Health | 1.066 | 1.023 | 1.070 | 1.026 | 1.029 | 0.752 | 0.978 | 1.026 | 1.070 | 1.046 | 0.983 | 1.029 | 0.934 | 0.953 | 1.045 | 1.085 |
| HIV/AIDS | 0.961 | 1.050 | 1.073 | 0.893 | 0.911 | 0.890 | 1.027 | 1.088 | 0.985 | 1.030 | 1.003 | 0.918 | 1.000 | 0.917 | 1.023 | 0.950 |
| Humanitarian financing | 0.848 | 1.097 | 0.938 | 0.929 | 0.901 | 1.031 | 0.968 | 1.069 | 1.050 | 0.976 | 0.872 | 0.983 | 0.952 | 0.968 | 1.006 | 0.985 |
| Logistics and telecommunications | 0.979 | 0.931 | 0.958 | 1.045 | 1.087 | 0.956 | 0.955 | 0.909 | 0.951 | 1.076 | 1.019 | 1.008 | 1.006 | 1.092 | 0.998 | 1.018 |
| Mine action | 0.891 | 0.939 | 1.135 | 0.926 | 0.853 | 0.979 | 0.992 | 0.869 | 0.931 | 1.067 | 0.957 | 1.086 | 1.012 | 0.905 | 0.975 | 1.104 |
| Peacekeeping and peacebuilding | 1.023 | 1.027 | 0.888 | 0.756 | 0.906 | 1.069 | 0.903 | 0.774 | 1.071 | 1.066 | 0.808 | 0.963 | 0.792 | 0.948 | 1.115 | 1.148 |
| Protection and human rights | 1.218 | 1.110 | 0.995 | 0.864 | 0.972 | 0.978 | 0.938 | 1.017 | 0.947 | 0.986 | 0.811 | 0.980 | 0.828 | 0.959 | 1.055 | 1.035 |
| Recovery and reconstruction | 0.966 | 1.001 | 1.046 | 0.869 | 0.962 | 1.118 | 0.948 | 0.989 | 0.978 | 0.969 | 0.987 | 0.947 | 0.976 | 1.014 | 0.949 | 1.039 |
| Safety and security | 1.088 | 1.014 | 1.105 | 0.907 | 1.030 | 0.930 | 0.911 | 0.949 | 0.998 | 0.858 | 0.831 | 0.992 | 0.971 | 1.023 | 0.995 | 1.040 |
| Shelter and non-food items | 1.000 | 0.994 | 0.997 | 1.127 | 1.035 | 1.018 | 1.025 | 0.974 | 0.973 | 0.932 | 0.996 | 1.004 | 0.949 | 1.136 | 0.861 | 1.036 |
| Water sanitation hygiene | 0.998 | 0.719 | 0.967 | 1.151 | 1.054 | 0.997 | 1.005 | 1.006 | 1.007 | 1.023 | 1.119 | 1.006 | 1.105 | 1.005 | 0.997 | 1.054 |

Table A. 4 Cause and effect distance correlation

| Reason  Sector | Cold wave | Drought | Earthquake | Epidemic | Extratropical cyclone | Fire | Flash flood | Flood | Heat wave | Insect infestation | Land slide | Mud slide | Other | Severe local storm | Snow avalanche | Storm surge | Technological disaster | Tropical cyclone | Tsunami | Volcano | Wild fire |
| --- | --- | --- | --- | --- | --- | --- | --- | --- | --- | --- | --- | --- | --- | --- | --- | --- | --- | --- | --- | --- | --- |
| Agriculture | 0.954 | 0.821 | 1.120 | 1.041 | 0.996 | 1.074 | 0.949 | 0.997 | 1.006 | 0.788 | 0.963 | 0.966 | 0.902 | 0.961 | 0.994 | 1.055 | 1.065 | 0.976 | 1.037 | 1.012 | 1.052 |
| Climate change and environment | 0.917 | 0.891 | 1.079 | 1.038 | 1.063 | 1.020 | 0.946 | 1.005 | 0.956 | 0.931 | 0.894 | 0.934 | 0.936 | 0.908 | 0.997 | 0.905 | 0.885 | 1.010 | 0.978 | 0.927 | 0.884 |
| Contributions | 0.998 | 1.024 | 0.979 | 1.007 | 0.913 | 0.945 | 1.031 | 0.985 | 0.948 | 0.924 | 1.002 | 0.972 | 1.060 | 1.046 | 1.010 | 0.968 | 0.941 | 0.968 | 0.988 | 0.987 | 0.979 |
| Coordination | 0.980 | 1.069 | 0.954 | 1.024 | 0.951 | 0.997 | 1.006 | 1.019 | 0.923 | 0.945 | 1.008 | 0.951 | 1.056 | 0.970 | 0.948 | 1.008 | 0.965 | 0.962 | 0.923 | 0.962 | 0.886 |
| Disaster management | 0.960 | 0.986 | 1.002 | 0.992 | 1.024 | 1.078 | 0.905 | 0.990 | 1.039 | 1.106 | 0.884 | 0.923 | 0.963 | 0.886 | 1.030 | 0.885 | 0.931 | 0.954 | 0.996 | 0.953 | 0.989 |
| Education | 0.982 | 1.006 | 0.990 | 0.932 | 0.994 | 1.027 | 0.994 | 1.034 | 1.062 | 0.990 | 0.998 | 0.992 | 0.958 | 1.051 | 1.028 | 0.938 | 1.006 | 1.018 | 0.987 | 0.953 | 1.172 |
| Food and nutrition | 0.993 | 0.913 | 1.060 | 1.076 | 1.045 | 0.952 | 0.978 | 0.985 | 1.025 | 0.967 | 0.994 | 0.961 | 0.962 | 1.007 | 0.986 | 1.022 | 1.054 | 1.003 | 1.057 | 0.995 | 1.004 |
| Gender | 0.978 | 0.876 | 0.958 | 1.021 | 0.851 | 0.934 | 1.072 | 1.021 | 0.915 | 1.097 | 0.997 | 0.914 | 0.919 | 1.009 | 0.963 | 1.034 | 0.973 | 1.014 | 0.976 | 0.969 | 0.942 |
| Health | 1.014 | 1.071 | 1.013 | 0.742 | 0.977 | 0.898 | 1.013 | 1.030 | 0.924 | 0.988 | 1.020 | 0.957 | 1.057 | 1.020 | 0.970 | 1.029 | 0.997 | 1.009 | 1.019 | 0.994 | 0.953 |
| HIV/AIDS | 0.951 | 0.916 | 0.966 | 0.933 | 1.008 | 0.947 | 0.977 | 0.996 | 0.986 | 0.963 | 1.007 | 0.977 | 0.916 | 1.067 | 0.932 | 0.957 | 1.069 | 0.887 | 0.980 | 1.057 | 0.961 |
| Humanitarian financing | 1.010 | 0.939 | 0.953 | 0.953 | 1.002 | 0.912 | 1.015 | 1.033 | 0.971 | 0.947 | 0.961 | 0.914 | 0.936 | 0.947 | 0.891 | 1.020 | 1.015 | 1.000 | 1.057 | 1.033 | 0.954 |
| Logistics and telecommunications | 1.021 | 0.936 | 1.068 | 0.935 | 1.038 | 1.164 | 0.844 | 1.014 | 1.087 | 0.979 | 0.879 | 1.067 | 0.856 | 1.054 | 0.969 | 0.792 | 1.046 | 0.941 | 1.106 | 1.026 | 1.021 |
| Mine action | 0.923 | 0.904 | 1.109 | 1.076 | 0.960 | 0.923 | 0.897 | 0.979 | 1.026 | 0.982 | 0.944 | 0.936 | 0.930 | 0.962 | 0.905 | 1.010 | 0.877 | 1.085 | 1.009 | 0.936 | 1.001 |
| Peacekeeping and peacebuilding | 1.030 | 0.757 | 1.022 | 0.905 | 1.008 | 1.016 | 1.030 | 0.995 | 0.963 | 1.018 | 0.901 | 0.955 | 0.752 | 1.103 | 0.979 | 0.988 | 0.878 | 1.075 | 1.036 | 1.113 | 1.008 |
| Protection and human rights | 1.035 | 0.934 | 1.059 | 0.934 | 1.071 | 0.988 | 0.955 | 1.010 | 0.982 | 1.029 | 0.986 | 0.948 | 0.828 | 1.021 | 0.993 | 1.010 | 0.983 | 1.071 | 0.992 | 0.917 | 1.028 |
| Recovery and reconstruction | 1.025 | 1.109 | 0.928 | 1.057 | 1.013 | 0.988 | 1.007 | 0.992 | 0.994 | 1.058 | 0.982 | 0.989 | 1.007 | 0.953 | 1.046 | 0.937 | 0.956 | 0.946 | 0.914 | 1.124 | 1.064 |
| Safety and security | 1.006 | 0.858 | 0.976 | 0.889 | 0.968 | 1.106 | 1.034 | 1.058 | 1.032 | 0.907 | 1.124 | 1.108 | 0.865 | 0.959 | 0.960 | 0.982 | 0.918 | 1.034 | 0.932 | 0.906 | 1.075 |
| Shelter and non-food items | 0.892 | 1.116 | 0.981 | 1.041 | 0.949 | 0.956 | 0.940 | 0.948 | 1.076 | 0.997 | 0.959 | 0.920 | 1.078 | 0.903 | 0.888 | 0.974 | 1.045 | 0.965 | 1.026 | 0.975 | 0.947 |
| Water sanitation hygiene | 0.991 | 0.964 | 1.032 | 0.980 | 0.983 | 0.976 | 0.988 | 0.985 | 1.038 | 1.103 | 0.992 | 0.983 | 1.012 | 1.050 | 1.055 | 1.044 | 1.040 | 0.983 | 1.029 | 0.965 | 1.067 |
